# ASpediaFI: Functional Interaction Analysis of Alternative Splicing Events

**DOI:** 10.1016/j.gpb.2021.10.004

**Published:** 2022-01-25

**Authors:** Kyubin Lee, Doyeong Yu, Daejin Hyung, Soo Young Cho, Charny Park

**Affiliations:** Bioinformatics Branch, Research Institute, National Cancer Center, Goyang 10408, Republic of Korea

**Keywords:** Alternative splicing, RNA-seq, Heterogeneous interaction network, Random walk with restart, Gene set enrichment analysis

## Abstract

**Alternative splicing** (AS) regulates biological processes governing phenotypes and diseases. Differential AS (DAS) gene test methods have been developed to investigate important exonic expression from high-throughput datasets. However, the DAS events extracted using statistical tests are insufficient to delineate relevant biological processes. In this study, we developed a novel application, Alternative Splicing Encyclopedia: Functional Interaction (ASpediaFI), to systemically identify DAS events and co-regulated genes and pathways. ASpediaFI establishes a **heterogeneous interaction network** of genes and their feature nodes (*i.e.*, AS events and pathways) connected by co-expression or pathway gene set knowledge. Next, ASpediaFI explores the interaction network using the **random walk with restart** algorithm and interrogates the proximity from a query gene set. Finally, ASpediaFI extracts significant AS events, genes, and pathways. To evaluate the performance of our method, we simulated RNA sequencing (**RNA-seq**) datasets to consider various conditions of sequencing depth and sample size. The performance was compared with that of other methods. Additionally, we analyzed three public datasets of cancer patients or cell lines to evaluate how well ASpediaFI detects biologically relevant candidates. ASpediaFI exhibits strong performance in both simulated and public datasets. Our integrative approach reveals that DAS events that recognize a global co-expression network and relevant pathways determine the functional importance of spliced genes in the subnetwork. ASpediaFI is publicly available at https://bioconductor.org/packages/ASpediaFI.

## Introduction

Alternative splicing (AS) is a key regulatory mechanism that confers protein diversity and plays an important role in inducing specific phenotypes and even causing diseases [1]. Wide-spread splicing events regulate distinct biological processes through gene interactions or controlling signaling of downstream genes [2], [3]. For instance, AS events induced by the splicing factor (SF) ESRP1 promote epithelial–mesenchymal transition (EMT) pathway activation [4], [5], [6]. In another case, the aberrant splicing of the epigenetic regulator *EZH2* induces large-scale differential expression associated with tumorigenic function [7]. To sum up such splicing cases, the cascade of spliceosome regulation induces distinct pathway activation or disease [7], [8], [9]. There are several difficulties in identifying the pathways induced by splicing genes using current methods. Novel statistical models have been developed to precisely detect differential alternative splicing (DAS) [10], [11], [12], [13], [14], [15]. However, multiple testing problems associated with testing thousands of whole transcriptomes still accompany the burden of false-positive errors [16], [17]. In addition, *P* values or ranks calculated by the DAS test did not provide any biological importance.

To investigate biological processes or pathways induced by the spliceosome, recent studies have extensively employed multi-layered transcript measures of both AS and gene expression profiles [8], [9]. The Cancer Genome Atlas (TCGA) and other cancer studies have investigated pathway activation characteristics induced by SF variants through gene set enrichment analysis (GSEA). The analyses were performed using gene expression profiles because reference gene sets have been developed for gene-level expression analysis [9], [18], [19], [20], [21]. In previous studies, two independent tests for differentially expressed gene (DEG) and DAS identification were performed. Its limitation is that co-operations between AS and genes are unidentifiable. Therefore, an integrative analysis approach is required to understand the comprehensive regulation of AS events and genes cascading from spliceosome regulation.

DAS promotes differential gene expression and specific biological processes. This implies a guilt-by-association between AS events and genes. SpliceNet and transcriptome-wide networks are appropriate approaches for establishing a co-expression network by splicing [22], [23]. However, these studies applied the isoform-centric method to employ isoform-level expression correlations. Although the isoform-centric approach successfully facilitates the inference of global associations between two splicing entities, it is difficult to identify precise exon usage and its genomic coordinates from isoforms. Consequently, this limitation could become an obstacle to the investigation of *cis*-regulatory elements or protein domain usage around the spliced exons. Currently, the splicing-centric methods, such as SeqGSEA and HBA-DEALS, integrate both DEG and DAS. These methods test AS genes to determine the pathways these genes are involved in [21], [24]. These approaches target quantitative integration of both the splicing and expression profiles of a gene. However, these methods did not infer co-regulation between splicing events and neighboring genes.

A heterogeneous network could be an appropriate model encompassing AS events, genes, and pathways to analyze cooperative regulation by splicing. In general, a heterogeneous network is an expansion of the interaction network that comprises nodes of different features. Several studies have considered such networks to integrate novel features such as genes, proteins, pathways, or diseases [25], [26], [27]. Edges of the heterogeneous network were weighted by various measures such as expression correlation, sequence homology, or pathway gene set to follow guilt-by-association [27]. Next, to identify the specific regulation module from the network, an important node should be extracted using a network traverse algorithm. The random walk with restart (RWR) algorithm was developed for an internet search engine to explore a web page network and has been extensively used to explore the biological interaction network [25], [27]. The algorithm traverses the network from a query and moves iteratively from nodes to neighboring nodes. Finally, it scores all the nodes based on proximity to the query. RWR has been successfully applied for creating disease–gene, protein–protein, or other heterogeneous interaction networks such as bipartite graphs [25], [27].

Here, we presented a novel method, Alternative Splicing Encyclopedia: Functional Interaction (ASpediaFI), to systematically identify AS events, co-expressed genes, and pathways involved using the RWR algorithm. We established a heterogeneous network composed of AS events, genes, and pathways as nodes, with edges weighted by the associations of percent spliced-in (PSI) with expression or gene–pathway associations. The RWR algorithm was used to extract the important nodes. Our application explores a heterogeneous interaction network using the discriminative random walk with restart (DRaWR) method [25]. We used a simple identifiable DEG set as the query. Finally, our method traverses the heterogeneous network and ranks all nodes to delineate splicing-associated regulation. The performance of our method was evaluated using simulated datasets and compared with those of the three DAS analysis tools. We applied our method to three RNA sequencing (RNA-seq) datasets involved in SF deficiency and demonstrated its biological relevance by referring to various studies. ASpediaFI is available in Bioconductor (https://bioconductor.org/packages/ASpediaFI).

## Method

### ASpediaFI workflow and dataset preparation

**Figure 1:**
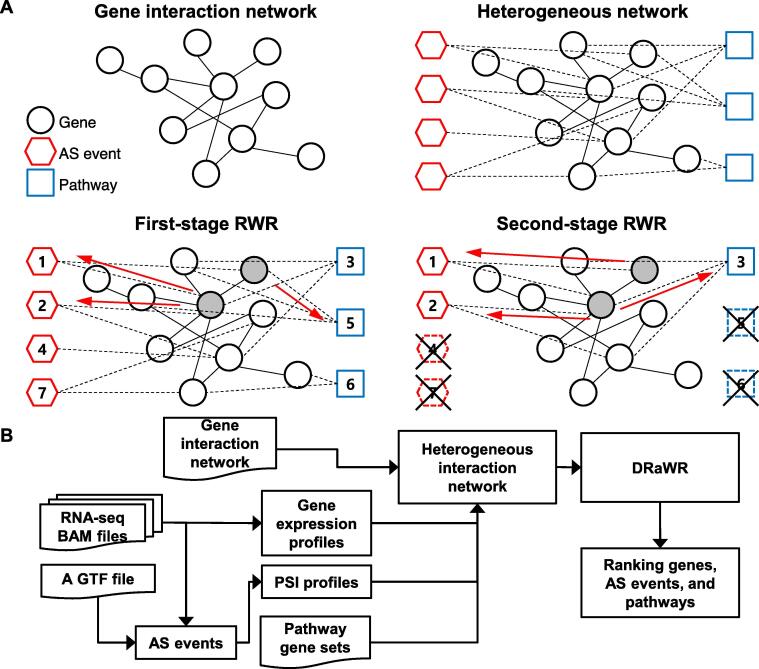
**ASpediaFI procedure** **A.** ASpediaFI extends the known gene interaction network and establishes a heterogeneous network for genes, AS events, genes, and pathways. Next, the method extracts relevant nodes from the two-stage RWR (see Method). **B.** The workflow illustrates that ASpediaFI requires input files, including the RNA-seq dataset, to establish a heterogeneous network, and exploits DRaWR to extract the final ranked result of query-relevant nodes. ASpediaFI, Alternative Splicing Encyclopedia: Functional Interaction; AS, alternative splicing; RWR, random walk with restart; DRaWR, discriminative RWR; RNA-seq, RNA sequencing; PSI, percent spliced-in.

ASpediaFI constructs a heterogeneous network for AS, gene, and pathway features (Figure 1A). First, the global gene interaction network (Figure 1A, upper left) is expanded by AS event and pathway nodes (Figure 1A, upper right). Next, the DRaWR algorithm traverses the network from a query gene set through a two-stage process and finds the proximity of each node from the query set [25]. DRaWR performs a two-stage RWR to select more relevant AS events and pathways. The first stage starts with a query gene set (Figure 1A, lower left; gray nodes), and the RWR assigns ranks for each node. In the second stage, low-ranking feature nodes are removed, and RWR finally re-ranks the retained nodes from the subnetwork (Figure 1A, lower right). Additionally, ASpediaFI performs a permutation test from randomly selected query sets to eliminate bias based on the size of the initial query set. Here, we employ the DEG set as a query to extract proximate AS event nodes as DAS. Finally, all nodes are scored by the stationary probability after RWR. The AS event, gene, and pathway nodes are simultaneously extracted.

In the data preparation step, ASpediaFI requires input files, including RNA-seq BAM files, a GTF format file for gene models, gene expression profiles, pathway gene sets, and a global gene–gene interaction network (Figure 1B). First, we generated a human gene interaction network reference collected from various interaction databases (BIND, DIP, HPRD, and REACTOME) [28], [29], [30], [31]. We also referred to pathway gene sets collected from public databases (Hallmark, REACTOME, and KEGG) and obtained 910 human pathway gene sets [20], [31], [32]. The gene expression profile was prepared from the FPKM results of RSEM or other quantification tools [33]. Next, ASpediaFI identified AS events from the gene model, and the events were classified into the following five types: alternative 5′ splice site (A5SS), alternative 3′ splice site (A3SS), skipping exon (SE), mutually exclusive exon (MXE), and retained intron (RI). The PSI (ψ) value for each AS event was calculated from the inclusion (I) and exclusion (E) values to sum the corresponding junction and exon read counts. We followed the rMATS method to calculate the isoform-level inclusion value I to divide by the effective length $l_{I}$, and the exclusion value E is divided by its effective length $l_{s}$ [10]. Finally, to indicate exon inclusion usage, PSI was calculated as $\psi =\left(I/l_{I}\right)/\left(I/l_{I}+E/l_{s}\right)$. The effective lengths $l_{I}$ and $l_{s}$ follow the equation of rMATS for each AS event type. Additionally, read counts were collected from fragment-level counts using paired-end sequencing. Our package refers to the functions of the IVAS package to follow the rMATS rule [10], [34]. Finally, we constructed a heterogeneous network based on the generated input files and performed DRaWR to explore the network. Reference information, such as gene interaction networks or pathway gene sets, can be altered for other organisms or novel databases in our tool.

### Establishment of an adjacency matrix for the heterogeneous network

The heterogeneous network comprises gene nodes and interacting feature nodes of AS events and pathways [25]. To generate an adjacency matrix for this network, we collect gene expression and PSI profiles from the RNA-seq dataset and refer gene–pathway associations from known pathway gene sets. Based on the prepared human reference network, we first establish the co-expressed gene–gene interaction network, where gene–gene interaction edges are weighted by the absolute value of the Pearson correlation coefficient calculated from gene expression (Figure 1A, upper left). Next, we connect gene nodes with feature nodes (AS events and pathways) based on prior information of gene–AS correlation passing a threshold and gene-pathway associations (Figure 1A, upper right). To take into account the non-monotonic rank relationship between two measures, gene expression and PSI, we connect gene–AS interaction edges if the absolute value of the Spearman correlation coefficient exceeds a user-defined threshold [35]. Finally, the gene–pathway edges are weighted to 1 if the corresponding gene belongs to the corresponding pathway gene set.

Let M be an initial adjacency matrix representing our heterogeneous network for each node: gene g, AS a, and pathway p. The adjacency matrix can be expressed as:

(1) $$M=\left[\begin{array}{ccc} M_{\mathrm{gg}} & M_{\mathrm{ga}} & M_{\mathrm{gp}}\\ M_{\mathrm{ag}} & M_{\mathrm{aa}} & M_{\mathrm{ap}}\\ M_{\mathrm{pg}} & M_{\mathrm{pa}} & M_{\mathrm{pp}} \end{array}\right]$$

where submatrices $M_{\mathrm{gg}}$, $M_{\mathrm{ga}}$, and $M_{\mathrm{gp}}$ exhibit a weight matrix between gene–gene, gene–AS, and gene–pathway, respectively. Therefore, the entries of M can be expressed as:

(2) $$m_{g_{i}g_{j}}=\left\{\begin{array}{c} {|r}_{P}\left(g_{i},g_{j}\right)|,\text{i}\text{f f}\text{o}\text{u}\text{n}\text{d i}\text{n t}\text{h}\text{e g}\text{e}\text{n}\text{e i}\text{n}\text{t}\text{e}\text{r}\text{a}\text{c}\text{t}\text{i}\text{o}\text{n n}\text{e}\text{t}\text{w}\text{o}\text{r}\text{k}\\ \;\;\;\;\;0,\text{o}\text{t}\text{h}\text{e}\text{r}\text{w}\text{i}\text{s}\text{e} \end{array}\right)$$

(3) $$m_{g_{i}a_{k}}=\left\{\begin{array}{c} {|r}_{S}\left(g_{i},a_{k}\right)\left|,\text{if}\;\text{|}r_{S}\left(g_{i},a_{k}\right)\right|>\tau \\ \;\;\;\;\;0,\text{o}\text{t}\text{h}\text{e}\text{r}\text{w}\text{i}\text{s}\text{e} \end{array}\right)$$

(4) $$m_{g_{i}p_{l}}=\left\{\begin{array}{c} 1,\text{i}\text{f a g}\text{e}\text{n}\text{e i}\text{s a m}\text{e}\text{m}\text{b}\text{e}\text{r o}\text{f a p}\text{a}\text{t}\text{h}\text{w}\text{a}\text{y g}\text{e}\text{n}\text{e s}\text{e}\text{t}\;\\ 0,\text{o}\text{t}\text{h}\text{e}\text{r}\text{w}\text{i}\text{s}\text{e} \end{array}\right)$$

where $r_{P}$ and $r_{S}$ are the Pearson and Spearman correlation coefficients, respectively, and τ is a user-defined threshold. Note that $M_{\mathrm{aa}}$, $M_{\mathrm{ap}}$, $M_{\mathrm{pa}}$, and $M_{\mathrm{pp}}$ are all zero matrices, as there are no edges among feature nodes. There is no connection between the AS and pathway feature nodes owing to the lack of evidence. Before running the RWR, we normalize our adjacency matrix M to derive a transition probability matrix, T [25]. First, we normalize for each non-zero submatrix $M_{\mathrm{XY}}$ to sum to one:

(5) $${\left(N_{X,Y}\right)}_{i,j}=\frac{{\left(M_{\mathrm{XY}}\right)}_{i,j}}{\sum_{i,j}{\left(M_{\mathrm{XY}}\right)}_{i,j}}$$

It equalizes the global probability of edges within features where the algorithm walks. Next, we normalize each column of matrix N, to compute a final transition probability matrix T.

(6) $$T_{i,j}=\frac{N_{i,j}}{\sum_{i}N_{i,j}}$$

It becomes the probability that the algorithm walker will take to transit from node j to i.

### Exploring the heterogeneous network and identifying query-specific important nodes by DRaWR

To extract the important nodes in our heterogeneous network, we employ DRaWR, a modified version of the RWR [25]. The DRaWR algorithm performs two-stage RWR. The first stage of RWR traverses all network nodes and scores the probability of nodes by a random walk (Figure 1A, lower left; red arrow). Before the second stage, low-ranked feature nodes from the first stage are eliminated, and the RWR is carried out on the subnetwork (Figure 1A, lower right) [25]. The second stage improves the ranking by performing RWR on a more relevant subnetwork [25]. Given a transition matrix T, the RWR algorithm can be formulated as:

(7) $$\pi^{t+1}=\left(1-c\right)T\pi^{t}+cv$$

where $\pi_{i}^{t}$ is the probability that the walker will stay at node i after the t-th iteration, c is the probability of restart, and $v_{j}$ is the probability of restarting at node j. That is, for a query gene set Q, $v_{j}$ is $\frac{1}{\left|Q\right|}$ if j∈Q, and zero otherwise. We assume $\pi^{0}$ to be a uniform probability vector such that $\pi_{i}^{0}=\frac{1}{n}$, where n is the number of all nodes in a heterogeneous network. The RWR with the query set is iterated until the vector $\pi^{t}$ converges ($|\pi^{t+1}-\pi^{t}|<0.05$). The converged $\tilde{\pi }$ represents the stationary probabilities (stat-*P* values) for all nodes. In stage 1 (Figure 1A, lower left), two RWRs run from a query gene set Q and another background query set B using all genes. The difference between the stat-*P* values in the two runs, say ${\tilde{\pi }}_{Q}-{\tilde{\pi }}_{B}$, is a final measure of relevance to a query gene set and is used to rank AS event nodes and pathway nodes altogether.

Before stage 2, ASpediaFI extracts a query-specific subnetwork composed of gene nodes and a user-defined number of highly ranked AS event nodes and pathway nodes. The adjacency matrix of the subnetwork can be expressed as:

(8) $$M\text{'}=\left[\begin{array}{ccc} M_{\mathrm{gg}} & M_{ga\text{'}} & M_{gp\text{'}}\\ M_{a\text{'}g} & M_{a\text{'}a\text{'}} & M_{ap\text{'}}\\ M_{p\text{'}g} & M_{p\text{'}a\text{'}} & M_{p\text{'}p\text{'}} \end{array}\right]$$

where a' and p' denote the nodes of the AS events and pathways retained in the subnetwork, respectively. M' is re-normalized as in the previous step and converted to a new transition probability matrix T'. The second stage of RWR repeats on the subnetwork T' to calculate stat-*P* which is similar to the first stage of RWR and produces final rankings of all nodes for AS events, genes, and pathways.

### Adjusting probabilities by a permutation test

To reduce the background effect caused by the size of the query gene set, we employ a permutation test for gene nodes as an additional procedure [36]. As previously conducted in the GSEA test [37], [38], we use a Monte Carlo method in which the second-stage RWR procedure is repeated *N* times with a randomly sampled query gene set of the same size as the actual query gene set. In each iteration, the randomized query gene set is used as the restart set, and the stationary probabilities for all gene nodes are measured as we do with the actual data. The permutation *P* value of gene node i is calculated as follows:

(9) $$P_{i}^{\mathrm{perm}}=\frac{1}{N}\sum_{n=1}^{N}I\left({\hat{\theta }}_{i}^{n}>{\hat{\pi }}_{i}\right)$$

where ${\hat{\theta }}_{i}^{n}$ is the second-stage stat-*P* of node i when a randomly sampled gene set is given as a query, and I is an indicator function that gives 1 if ${\hat{\theta }}_{i}^{n}>{\hat{\pi }}_{i}$ and 0 otherwise. This procedure determines whether the observed stationary probabilities for gene nodes are statistically significant relative to random query gene sets. Finally, to remove the bias by query gene set size, we measure the permutation *P* value for 1000 iterations and extract statistically significant gene nodes ($P_{i}^{\mathrm{perm}}$ < 0.05) for the final AS-relevant subnetwork.

### Performance benchmark using simulated datasets

We evaluated the performance of ASpediaFI for DAS detection against other state-of-the-art methods using simulated datasets. To simulate datasets close to a realistic setting, we referred to the real RNA-seq dataset from myelodysplastic syndrome (MDS) patients [19]. Two groups [*SF3B1* mutated (MUT) and wild-type (WT)] of RNA-seq reads were generated using Flux Simulator, and we followed the simulation pipeline previously developed [39], [40]. We referred to the GRCh38 GENCODE v31 gene model and simulated 20, 10, and 5 replicates per condition, as well as five levels of mean base coverage (150×, 65×, 50×, 30×, and 15×) for 100 bp paired-end reads. In the first step of the simulation pipeline, gene-level read counts were produced based on the MDS dataset for each group. The read counts were simulated to follow the negative binomial (NB) distribution for modeling the variance across biological replicates. Next, we randomly chose 1000 DAS genes between the conditions. For these 1000 genes serving as the ground truth for the simulated dataset, we set relative isoform abundances such that the last isoform took a predetermined proportion (0.8, 0.6, and 0.4 for MUT and 0.2 for WT), and other isoforms equally shared the rest. The isoform-level abundance of non-DAS genes was drawn from a uniform distribution. Each fastq file was mapped using the STAR v2.5.1b [41]. Overall, we artificially generated RNA-seq BAM files of varying sample sizes and sequencing depths to evaluate the discriminative power and robustness of the method.

For each simulated dataset of different sample sizes and sequencing depths, we performed DAS analysis and compared ASpediaFI with three other widely used event-based methods: MISO v0.5.4, rMATS v4.0.2, and SUPPA2 v2.3 [10], [11], [12]. For all methods under comparison, AS events were derived from the GENCODE v31 gene model. For ASpediaFI, we first quantified the gene expression profile using the RSEM v1.3.0 [33]. The PSI profiles of the BAM files were calculated using the IVAS package following the rMATS rule [10], [34]. To extract DAS nodes from the established network, we defined a query set from DEGs using limma v3.42.0 [log_2_ fold change (FC) > 2; adjusted *P* value < 0.001] because the RWR algorithm starts at the given DEG nodes and traverses the prior network to retrieve correlated AS feature nodes [42]. Then, the ASpediaFI analysis was run with the following options: restart (restart probability) = 0.7, number of folds (number of folds for cross-validation) = 5, number of features to be retained in a subnetwork = 1000, low.expr (threshold FPKM average of genes) = 1, low.var (threshold variance of AS events) = NULL, prop.na (threshold proportion of missing PSI values) = 0.05, prop.extreme (threshold proportion of extreme PSI values – 0 or 1) = 1, cor.threshold (threshold Spearman’s correlation coefficient between genes and AS events) = 0.4, 0.5, or 0.6.

For MISO, as only pairwise comparisons were allowed in the DAS analysis, we merged BAM files into one pooled data per condition. DAS analysis was performed using the pooled version of the BAM files, and other parameters were used in the default settings. rMATS was run using the default settings. For SUPPA2, to obtain PSI profiles, we quantified transcript expression in the TPM units using RSEM. Next, we executed the embedded modules psiPerEvent to generate PSI profiles and diffSplice to detect DAS events using default options.

To evaluate the discriminative power of the four methods, we generated a receiver operating characteristic (ROC) curve and computed the area under the curve (AUC) metric using the R PRROC package [43]. The same number of highly ranked DAS events (top 1000) was extracted from each method’s results for a fair comparison. ASpediaFI yields rankings for nodes of a previously defined size as the final optimal output after the second-stage RWR. Therefore, the most significant 1000 DAS events for the other three methods were assessed to make a fair comparison. The ranking of AS events was determined based on adjusted *P* values for rMATS and SUPPA2, Bayes Factor (BF) for MISO, and stat-*P* for ASpediaFI. Moreover, we used the AUC metric to assess the effects of the sample size and sequencing depth.

### Case study 1: MDS patients

In the first case study, we used an RNA-seq dataset (GEO: GSE114922) from bone marrow-derived CD34^+^ hematopoietic progenitor cells from 82 patients with MDS [19]. Patients exhibited hotspot mutations in three SFs: *SF3B1* (n = 28), *SRSF2* (n = 6), and *U2AF1* (n = 8). We first assessed the quality of reads using FastQC v0.11.5, and aligned them to the GRCh38 genome and the reference gene model GENCODE v31 via STAR v2.6.1b, following the GDC pipeline with customized options: outFilterType = BySJout, alignEndsType = EndToEnd, alignSoftClipAtReferenceEnds = No, alignIntronMax = 10,000, and alignMatesGapMax = 10,000 [41].

We extracted DAS for each SF condition using ASpediaFI, and compared its performance with those of the other three methods used in the previous simulation analysis. For ASpediaFI, the query gene set was obtained from a DEG analysis between the SF MUT and WT samples using limma. We employed DEGs (adjusted *P* value < 0.001; log_2_ FC > 1) as a query (n = 112). To evaluate the performance with respect to the query set, we also assessed various queries for DEGs (adjusted *P* value < 0.001; log_2_ FC > 0.6 or 0.4), pathway gene sets associated with biological relevance (heme metabolism, n = 200), and randomly chosen genes (n = 100). Given the query gene set, ASpediaFI was run with the following options: restart = 0.7, num.folds = 5, num.feats = 300, low.expr = 1, low.var = NULL, prop.na = 0.05, prop.extreme = 1, and cor.threshold = 0.4. Based on the second stage result of DRaWR, we reconstructed an AS–gene interaction subnetwork regulated by each SF mutation. Additionally, genes (permutation *P* < 0.05) were selected along with neighboring AS event nodes (Table S1). For the other three tools under comparison, we applied the same options as described in the previous simulation analysis. To reflect the characteristics of each tool, we imposed additional filtering conditions. Following a distinct MISO analysis strategy, we generated a pooled BAM file for each condition. Due to the large sample size being pooled, we decided to apply more stringent BF and minimal coverage than default settings (BF ≥ 300, the sum of inclusion and exclusion reads ≥ 300, 100, and 80, at least 30, 10, and 8 inclusion and exclusion reads for *SF3B1*, *SRSF2*, and *U2AF1*, respectively). A conventional cutoff [false discovery rate (FDR) < 5%] was applied to the SUPPA2 results. Meanwhile, the rMATS analysis results were filtered with a stricter threshold (FDR < 0.1%). Like MISO, we ought to apply stringent thresholds on rMATS results because the conventional cutoff was non-discriminative, yielding a substantially large number of resultant DAS events.

We evaluated the results in terms of various aspects. First, we investigated whether our results detected the actual DAS. ASpediaFI does not directly perform the DAS test and identifies AS events from the proximity of the query gene set. From the *SF3B1* comparison case, we extracted the PSI profile of the resultant AS events. We then performed hierarchical clustering with complete linkage on the Euclidean distance of the PSI profile to classify samples and tested its performance (sensitivity and specificity) to discriminate between *SF3B1* MUT and WT samples. Next, we examined the motif sequence associated with the splicing site in the *SF3B1* case for *cis*-element level validation. We extracted the acceptor site sequence from a 35 bp exon upstream to 3 bp downstream of the A3SS events involved in *SF3B1* [18]. The motif was identified using the R package ggseqlogo [44], and A3SS DAS events were compared with non-DAS. Finally, we investigated the functional importance of AS events from additional sequence information such as protein domain, splicing-specific protein–protein interactions (PPIs), and nonsense-mediated decay (NMD). We explored the ASpedia database for each AS event and compared the results obtained using all four methods [45].

The distinct advantage of our method is that it identifies genes, AS events, and pathways. We demonstrate our method of determining how well AS-relevant pathways by comparing with GSEA using gene expression only. To conduct a GSEA test, we performed gene set variant analysis (GSVA) to estimate pathway activity for hallmark pathways [46]. The pathway activity difference for SF MUT was tested using the Wilcoxon rank-sum test. To evaluate how well our method detects DAS events involved in biologically relevant pathways, we carefully selected the gold standard, the heme metabolism (HM) pathway, based on multiple previous studies [47], [48], [49], [50], [51], [52], [53]. To compare the degree of HM enrichment for the DAS results of the four methods, we conducted Fisher’s exact test and computed the precision, recall, and F_1_ score. We also defined a novel HM gene set, ‘HM expansion set’, to assess whether AS events interact with HM pathway genes and participate in the corresponding biological process. The HM expansion set included both HM genes and their neighboring genes, derived from our gene interaction network. The same four metrics were computed for the HM expansion set.

### Case study 2: TCGA stomach adenocarcinoma

We chose the TCGA stomach adenocarcinoma (STAD) level 3 RNA-seq dataset as another real dataset to investigate AS events and biological processes associated with *ESRP1*, a key SF that regulates EMT across multiple cancer types [10], [22], [54]. Of the 415 STAD patients, the highest and lowest 10% mRNA expression samples of *ESRP1* were classified as *ESRP1*-high and *ESRP1*-low groups, respectively. Due to the absence of BAM files, we referred to a gene model of UCSC known genes and used SUPPA2 v2.3 to generate PSI profiles, as done in a previous study [55]. A DEG test between the two groups was performed using limma to obtain a query gene set (adjusted *P* value < 1.0E−8; log_2_ FC > 2). ASpediaFI analysis was conducted with the following options: restart = 0.7, num.folds = 5, num.feats = 300, low.expr = 1, low.var = NULL, prop.na = 0.05, prop.extreme = 1, and cor.threshold = 0.5. To compare our results, we performed DAS analysis using SUPPA2 diffSplice with the following options: nan-threshold = 10, area = 1000, and lower-bound = 0.05 [12]. The SUPPA2 DAS set was obtained by selecting AS events with $\left|\Delta PSI\right|$ > 0.1, and adjusted *P* value < 0.1. Next, we extracted an EMT-associated subnetwork from the final stage produced by DRaWR. To decrease the network size, we retained gene nodes with permutation *P* values less than 0.05.

Similar to case study 1 analysis to evaluate the discriminative power of ASpediaFI, we tested our resultant AS events by classifying STAD samples based on the Euclidean distance matrix of their PSI profiles, using hierarchical clustering with average linkage. Meanwhile, we assessed the extent to which our AS-relevant pathway results were consistent with GSEA using gene expression profiles. Our pathway results were collected based on the rankings determined by ASpediaFI. For GSEA, we calculated sample-level pathway activity scores and performed GSVA [46]. The difference in GSVA scores between the high and low groups was tested using the Wilcoxon rank-sum test. Additionally, we compared ASpediaFI with another DAS test method. The results from the two applications, ASpediaFI and SUPPA2, were compared using the Venn diagram, Fisher’s exact test, and Jaccard index based on the five EMT- or *ESRP1*-associated splicing gene signatures [4], [6], [56], [57]. As in case study 1, AS event sets for the two conditions were chosen to overlap with global PPI genes.

### Case study 3: *RBFOX1*-knockdown cell lines

The last RNA-seq dataset (GEO: GSE36710) comprised five replicates of the shRBFOX1 (*RBFOX1* knockdown) and shGFP (control) cell lines [58]. Single-end RNA-seq reads were aligned to the GRCh37 genome and the reference gene model Ensembl v71, using STAR v2.6.1b with the same options as in case study 1. We calculated gene expression and PSI values using RSEM and our quantification tool, respectively. A query gene set was obtained from the DEG test between *RBFOX1-*knockdown and control groups using limma (adjusted *P* value < 0.05). The following options were selected for ASpediaFI: restart = 0.7, num.folds = 5, num.feats = 300, low.expr = 1, low.var = NULL, prop.na = 0.05, prop.extreme = 1, and cor.threshold = 0.8. We then investigated an *RBFOX1*-regulated subnetwork. From the final network produced by the DRaWR algorithm, we retained gene nodes with permutation *P* values less than 0.05, and their neighboring AS event nodes. For the other three tools under comparison, we applied the same options described in case study 1, except for the type of read-option setting to single-end. Conventional cutoffs were applied to the results of MISO (BF > 10), rMATS (FDR < 10%), and SUPPA2 (FDR < 5%).

To examine AS gene enrichment in known neuronal genes, we calculated the Jaccard index between the AS gene sets of the four tools and known gene signatures, as done in a previous study [58]. We prepared seven published gene signatures containing genes inferred from the transcriptomic analysis of *RBFOX1* and *RBFOX2* and those showing *RBFOX1*-dependent splicing in brains with autism spectrum disorder [59], [60], [61], [62], [63], [64]. We also compared these with three control gene signatures: mitochondria, epilepsy, and ataxia [58]. To evaluate the performance of ASpediaFI in identifying biologically relevant pathways, we performed GSEA on gene nodes with permutation *P* values less than 0.05, using DAVID v6.8 [65]. Our GSEA results were compared with two previously identified gene sets: blue module and DAS [58]. The blue module comprising 737 genes is a subnetwork identified by WGCNA using gene expression profiles; DAS contains 603 events detected by DESeq [66].

## Results

### Performance evaluation on simulated datasets

To evaluate the performance of ASpediaFI for DAS event detection, we conducted a benchmarking analysis using simulated datasets of various conditions based on 1000 artificial DAS genes. The four event-based methods were tested on 15 simulated RNA-seq datasets of various sample sizes (n = 20, 10, and 5 replicates per condition) and mean base coverages (depth = 150×, 65×, 50×, 30×, and 15×). We generated RNA-seq datasets of the five different read counts (average read counts of approximately 52 million, 22 million, 17 million, 10 million, and 5 million, respectively) (Table S2).

**Figure 2:**
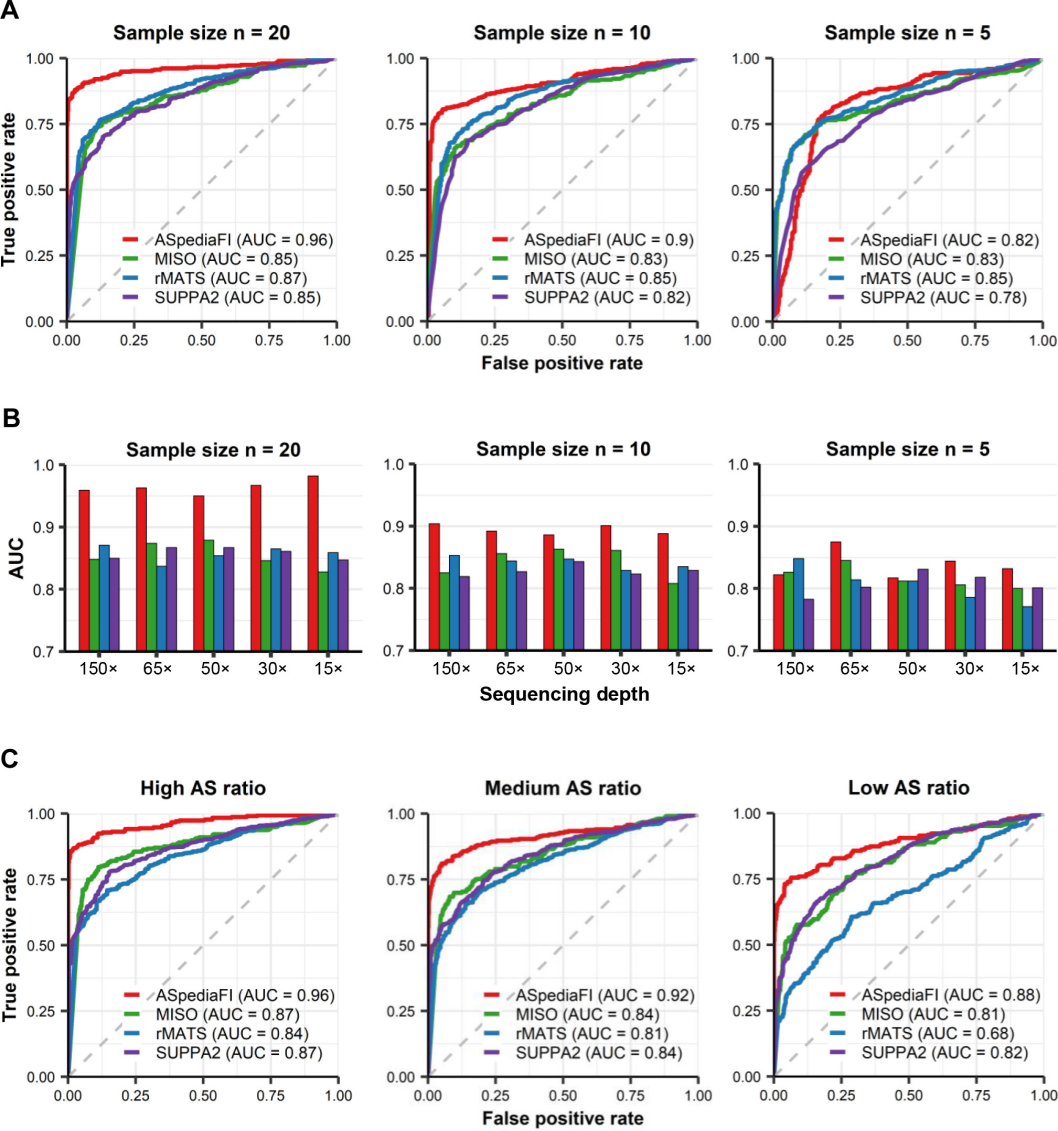
**Performance evaluation and comparison of ASpediaFI and three other methods on simulated RNA-seq datasets** **A.** ROC curves to evaluate the accuracy of four methods detecting DAS events from simulated datasets of mean base coverage (depth = 150×) and varying sample sizes (n = 20, 10, and 5 replicates per condition). ROC curves for each method illustrate the true-positive rate (y-axis) against the false-positive rate (x-axis). AUC values are described for each method. The dashed diagonal line corresponds to a ROC curve when DAS predictions are randomly guessed (AUC = 0.5). **B.** Bar plots of AUC for evaluating sequencing depth effect (depth = 150×, 65×, 50×, 30×, and 15×) and sample size effect (n = 20, 10, and 5 replicates per condition). **C.** ROC curves to compare the performance of four methods under three isoform usage conditions of high (0.8), medium (0.6), and low (0.4), with other conditions shared (sequencing depth 65× and sample size 20). ROC, receiver operating characteristic; DAS, differential alternative splicing; AUC, area under the curve.

We computed the ROC and AUC metrics to compare the performance of the four methods. Under the mean base coverage of 150×, ASpediaFI consistently achieved the highest AUC across various sample sizes, while exhibiting relatively competitive performance for the smallest dataset (n = 5; Figure 2A). In all four methods, the AUC values increased as the sample size increased from 5 to 20. ASpediaFI showed the best performance among the four methods (Figure 2A). Based on the AUC values, ASpediaFI exhibited the best performance across all sequencing depths in 20 replicates per condition (Figure 2A, Figure S1). Importantly, in the critical region with a false-positive rate between 0 and 0.25, ASpediaFI consistently achieved the highest true positive rate among the four methods, except for a sample size of 5 (Figure 2A, Figure S1).

When assessing the performance under various sequencing depth conditions, all four methods demonstrated stable performance under varying depths. Under the sample sizes of 20 and 10, all four methods robustly identified true DAS events with AUC values maintained around 0.8 or above, even if the sequencing depth decreased (Figure 2B). However, all four methods exhibited relatively lower discriminative power when the sequencing depth decreased unber the smallest sample size (n = 5). Specifically, in all four methods, AUC values decreased with the largest degree at the lowest depth (depth = 15×) and the smallest sample size (n = 5). The AUC value of rMATS was mostly decreased from 0.85 to 0.77, while the other three methods maintained an AUC value of at least 0.8 (Figure 2B, Figure S1). Despite the unsuitable condition (sequenicng depth 12× and sample size 5), ASpediaFI maintained higher AUC values than the other three methods (ASpediaFI = 0.83, MISO = 0.80, rMATS = 0.77, and SUPPA2 = 0.80). Moreover, ASpediaFI achieved consistently higher AUC statistics and showed the best discriminative power for sample sizes of 20 and 10 even when the sequencing depths varied (Figure 2B). Notably, under the lowest depth (depth = 15×) and largest sample size (n = 20), ASpediaFI demonstrated the best performance with the largest AUC difference (ASpediaFI = 0.98, MISO = 0.83, rMATS = 0.86, and SUPPA2 = 0.85). Overall, all four methods showed robust performance across various sequencing depths, except for the smallest sample size (n = 5). However, ASpediaFI consistently showed superior discriminative power in our simulation analysis.

Additionally, we compared the performance under three different isoform usage conditions for case samples (low = 0.4, medium = 0.6, and high = 0.8). Other factors (sequening depth 65× and sample size 20) were fixed with isoform usage (0.2) for control samples (Figure 2C). Our method showed the best performance (AUC = 0.88–0.96), compared to other methods (AUC = 0.68–0.87). Moreover, as ASpediaFI requires a correlation coefficient option for connecting network edges, we tested cutoffs (*r* = 0.4, 0.5, and 0.6) using datasets with sequencing depths of 150× and 65× (Table S3). For a fixed sequencing depth and sample size, AUC values were maintained across the cutoffs, ranging from moderate to strong correlation coefficients. When stricter cutoffs were applied to the largest dataset (n = 20), ASpediaFI performance consistently exhibited good performance, increasing up to AUC 0.97 for both sequencing depths (Table S3). Overall, ASpediaFI exhibited a robust performance under various AS ratios and correlation thresholds for network edges.

### AS analysis and evaluation from three RNA-seq datasets

To assess the performance of our method, we employed three RNA-seq datasets from MDS patients, STAD patients, and *RBFOX1*-knockdown cell lines, respectively. MDS and STAD were collected from a large-scale cancer dataset, and the *RBFOX1* set included two-condition samples of a relatively smaller size (n = 5 per condition). The datasets contain SF mutations or down-regulation which induce AS events. We performed ASpediaFI analysis and compared it with other methods. The DAS, pathway, and network topological details are summarized in Table S1. Various evaluations were performed based on the characteristics of three case studies.

#### *Case study 1*: *mutations in three SFs induce the dysregulation of heme metabolism in MDS*

We investigated AS events in RNA-seq samples from MDS patients (n = 82) induced by SF deficiency on *SF3B1* (n = 28), *SRSF2* (n = 8), and *U2AF1* (n = 6). Initially, a heterogeneous network contained 54,032 nodes and 108,074 edges for a gene–gene interaction network with Kleinberg’s hub centrality score average of 0.017. After filtering out low-quality genes and adding AS feature nodes, the network finally contained 6142 AS events with 326,532 gene–AS edges for the first-stage RWR. ASpediaFI was independently applied to these three SF-deficient MDS datasets using three query gene sets from DEGs (n = 112, 107, and 96) [19]. By comparing SF MUT with WT samples, ASpediaFI identified 281, 269, and 285 DAS events and 19, 31, and 15 pathways for *SF3B1*, *SRSF2*, and *U2AF1*, respectively (Table S1). For comparison with other methods, we extracted DAS events via MISO (n = 2119), rMATS (n = 2395), and SUPPA2 (n = 208). When we reduced the MISO and rMATS DAS events to approximately 200–300, similar to ASpediaFI or SUPPA2, only SE events were left. Therefore, it is unavoidable to extract over 2000 DAS events to detect five AS events. MISO and rMATS provided *de novo* splicing event analyses, so we additionally investigated whether novel events were being. Among the resultant DAS events, 21% and 6% were novel in MISO and rMATS, respectively. The number of known events for the two methods was still larger than that for ASpediaFI and SUPPA2. Next, we compared our results with other methods to evaluate how biologically relevant extracted AS events were. We evaluated the extent to which the performance is associated with various query conditions of strict DEG cutoffs and pathway gene sets. Finally, the *cis*-element motifs and functional sequences, such as protein domain and NMD, were investigated for further validation.

**Figure 3:**
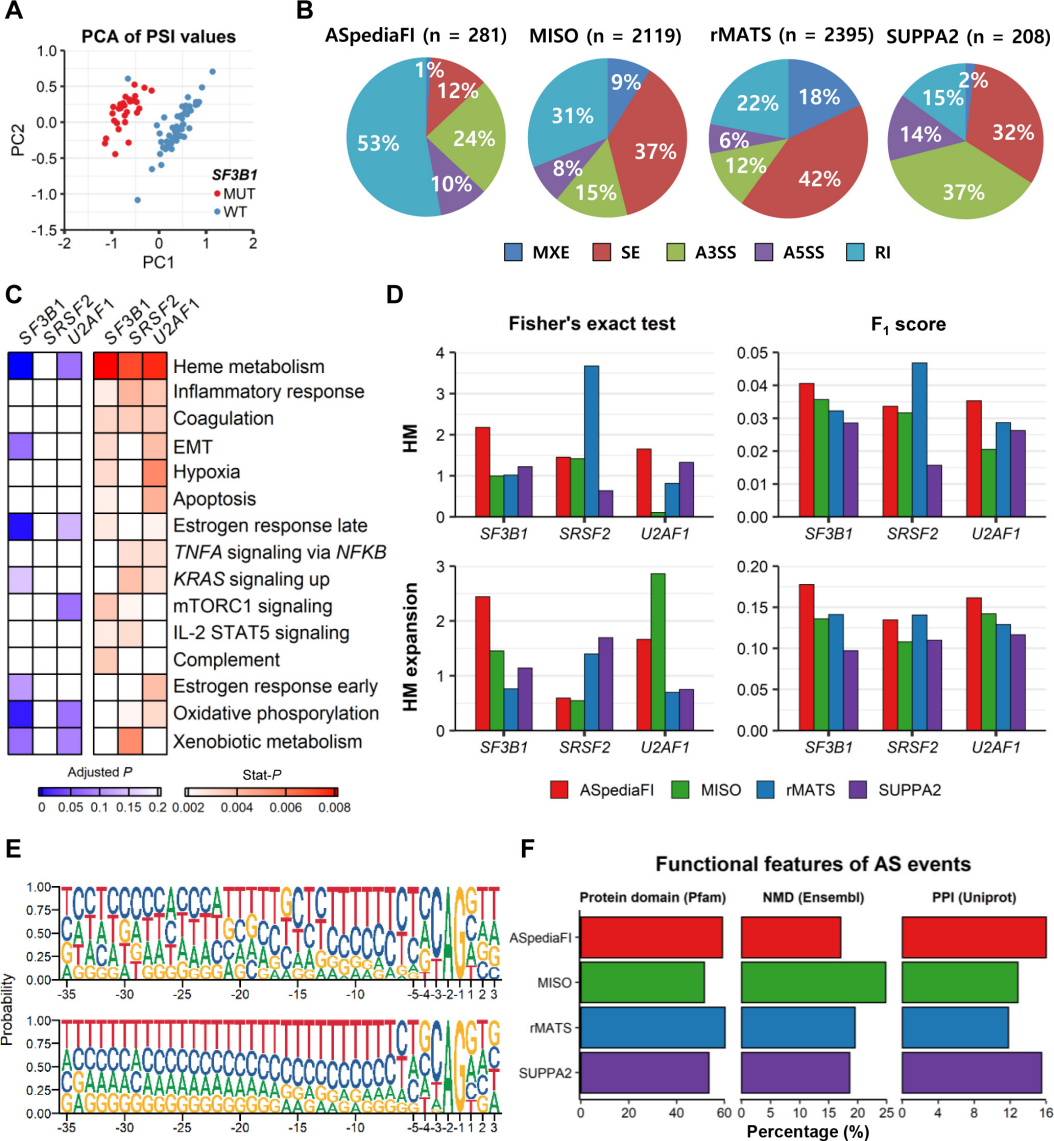
**Identification ofAS events and pathways regulated by *SF3B1*, *SRSF2*, and *U2AF1* mutationsin the MDS dataset** **A.** PCA plot derived from PSI values of 281 DAS events identified in *SF3B1* MUT samples. **B.** Percentages of five AS types identified by the four methods in the *SF3B1* MUT case. Total counts of identified DAS events are indicated at the top of each method’s pie chart. **C.** Enrichment comparison of pathways identified by gene expression-based analysis and ASpediaFI. The heatmap (right) represents the degree of enrichment in the top 15 pathways across the three SF analyses (ranked by ASpediaFI stat-*P* values). The selected pathways were tested in GSEA using gene expression profiles only. The heatmap (left) depicts the statistical significance of pathway-level GSVA scores. **D.** Bar plots of Fisher’s exact test *P* values (−log_10_) and F_1_ scores to test pathway enrichment for both HM and expansion sets. Enrichment was tested from two pathway gene sets and DAS event gene sets identified from four methods. The degree of enrichment in the two pathway gene sets was compared across the four methods and the three SF analyses. **E.** Sequence logos of the motif detected around the A3SS. Motifs from 35 nt upstream of the 3′ AG to 3 nt downstream are represented (DAS at the top and non-DAS at the bottom). **F.** Percentage bar plots of AS events to contain three functional sequence features: protein domain, NMD, and isoform-specific PPIs. These functional features are compared between the four methods. MDS, myelodysplastic syndrome; PCA, principal component analysis; PC1, principal component 1; PC2, principal component 2; MUT, mutated; SF, splicing factor; stat-*P*, stationary probability; GSEA, gene set enrichment analysis; GSVA, gene set variant analysis; HM, heme metabolism; A3SS, alternative 3′ splice site; NMD, nonsense-mediated decay; PPI, protein–protein interaction.

We demonstrated the performance of our method for identifying AS events. Contrary to other methods, our method provided distinct steps to exploit the DEG query and identify AS events co-expressed with the query gene set. Therefore, to evaluate the discriminative ability of our method, we extracted PSI profiles of the resultant AS events and tested their performance using a hierarchical clustering approach. As depicted in a scatterplot of principal component analysis (PCA) for the *SF3B1* case, our method accurately discriminated MUT and WT samples (sensitivity: 100%, specificity: 96.3%; Figure 3A).

Next, we examined the proportions of the five AS event types for the four methods (Figure 3B; Table S4). For ASpediaFI, RI (38%−53%) was frequently detected across the three SF analyses, followed by A3SS (Figure 3B, Figure S2). The dominant occurrence of A3SS and RI events resembles previous MDS studies [18], [19], [47], [48], [51], [52], [53]. rMATS and MISO detected SE as the dominant event type across the three SF analyses (rMATS: 42%−63%, MISO: 37%−53%; Table S4); rMATS detected RI as the second most frequent event type in the analyses of *SF3B1* and *U2AF1* (22% and 16%, respectively), while MISO detected RI as the second most frequent event type across the three SF analyses (15%−31%). While rMATS and MISO showed a similar pattern, SUPPA2 detected A3SS (37%) most frequently, followed by SE (32%) and RI (15%) in *SF3B1* analysis (Figure 3B). For other analyses of *SRSF2* and *U2AF1* using SUPPA2, SE took the most substantial proportion (44%−47%) and A3SS (20%−25%) as the second-largest type (Table S4). The top 200–300 ranked AS events exhibited a difference in AS type proportions in SE and RI events. Previous studies of the *SF3B1* variant to disrupt A3SS indicated that rMATS and MISO detected more proportions of SE-type events [18], [67]. Meanwhile, ASpediaFI and SUPPA2 detected A3SS events relatively well but presented a difference in the RI type proportion. For the other comprehensive SF MUT analyses, our AS-type counts resembled TCGA acute myeloid leukemia, lung cancer, and uveal melanoma in the A3SS type being dominant (Figure S2) [18], [19]. In contrast, rMATS and MISO detected SE events as the most frequent type in all three SF analyses.

A distinct advantage of our method is that it extracts interacting genes and pathways associated with AS event sets. We demonstrated the relevance of these pathway results using GSEA based on gene expression. We extracted highly ranked hallmark pathways using GSVA and limma from our three SF MUT analysis results (Figure 3C, right). The HM pathway was ranked highest in all three SF analyses. Additionally, the inflammatory response, coagulation, and hypoxia pathways were found to be regulated by the three mutated SFs. Our extracted pathways were notably consistent with a previous MDS analysis [19]. ASpediaFI also consistently detected HM, EMT, and estrogen receptor signaling pathways (Figure 3C, right), compared with the GSEA top-ranked pathways (Figure 3C, left).

We further evaluated the functional enrichment of DAS event sets detected by the four methods. Previous studies reported that disrupted splicing due to *SF3B1* or *U2AF1* mutation involves hematopoietic malignancy, HM, and heme biosynthesis in MDS or other blood cancers [47], [48], [49], [50], [51], [52], [53]. Therefore, we selected the HM pathway and its expansion set as criteria to evaluate performance. ASpediaFI demonstrated the highest enrichment of both HM and expansion sets in the *SF3B1* analysis in terms of Fisher’s exact test *P* values and F_1_ scores (Figure 3D). Although other methods, particularly rMATS, exhibited superior performance according to Fisher’s *P* values in the *SRSF2* analysis (HM set, *P* = 0.00021; HM expansion set, *P* = 0.039), F_1_ scores indicated that ASpediaFI achieved relatively reasonable performance in the *SRSF2* analysis (Figure 3D, Figure S3). In addition, our method showed the highest degree of HM and expansion enrichment based on the F_1_ scores in the *U2AF1* analysis (ASpediaFI: 0.035 and 0.16, rMATS: 0.028 and 0.13, respectively, for HM and expansion sets; Figure 3D). Because there is no gene set database for AS events, we defined HM pathway genes as true positive. Therefore, the overall F_1_ scores are low (< 0.2) compared with simulation analyses in which the ground truth is well defined. Next, we verified the performance based on multiple query gene sets. When testing various DEG queries (adjusted *P* value < 0.001; log_2_ FC > 1, 0.6, or 0.4), ASpediaFI showed the best F_1_ score under the strict DEG cutoff condition (Table S5). Querying the HM pathway showed almost the same performance as DEGs under the strict condition (adjusted *P* value < 0.001; log_2_ FC > 1), whereas the poorest result was obtained for the randomly selected gene query set (Table S5).

Additionally, we validated the performance of *cis*-elements to detect more A3SS in the previous *SF3B1* analysis. We contrasted motifs around acceptor sites between the identified DAS events (n = 66) and non-DAS events (n = 2469). As depicted in the motif frequency plots (Figure 3E), the upstream region of the canonical AG dinucleotide (−2 to −1 nt) was enriched with the long polypyrimidine (Py) tract (bottom plot); meanwhile, DAS events were observed with shorter Py tracts and multiple motifs at −16 to −13 nt and −35 to −22  nt regions, respectively (Figure 3E, top plot). In particular, the perturbation of *cis*-elements of regions −16 to −13  nt was consistent with previous *SF3B1* K700E mutation analysis investigating −20 to +3 nt regions [68]. Next, we explored various functional sequence features of splicing regions, such as the protein domain or NMD [45]. Although one feature, such as the protein domain, could not become an absolute measure to compare the performance, we could infer the functional importance of the detected results. Across the three SF analyses, our method presented more DAS events involved in protein domains (Pfam) and isoform-specific PPIs, compared to those identified by other methods (Figure 3F, Figure S4). MISO detected the most frequent NMD-associated AS events. Overall, there was no significant difference noted among the four methods according to the functional sequence features.

#### *Case study 2*: *EMT pathway in stomach cancer induced by ESRP1 and the representative AS events*

**Figure 4:**
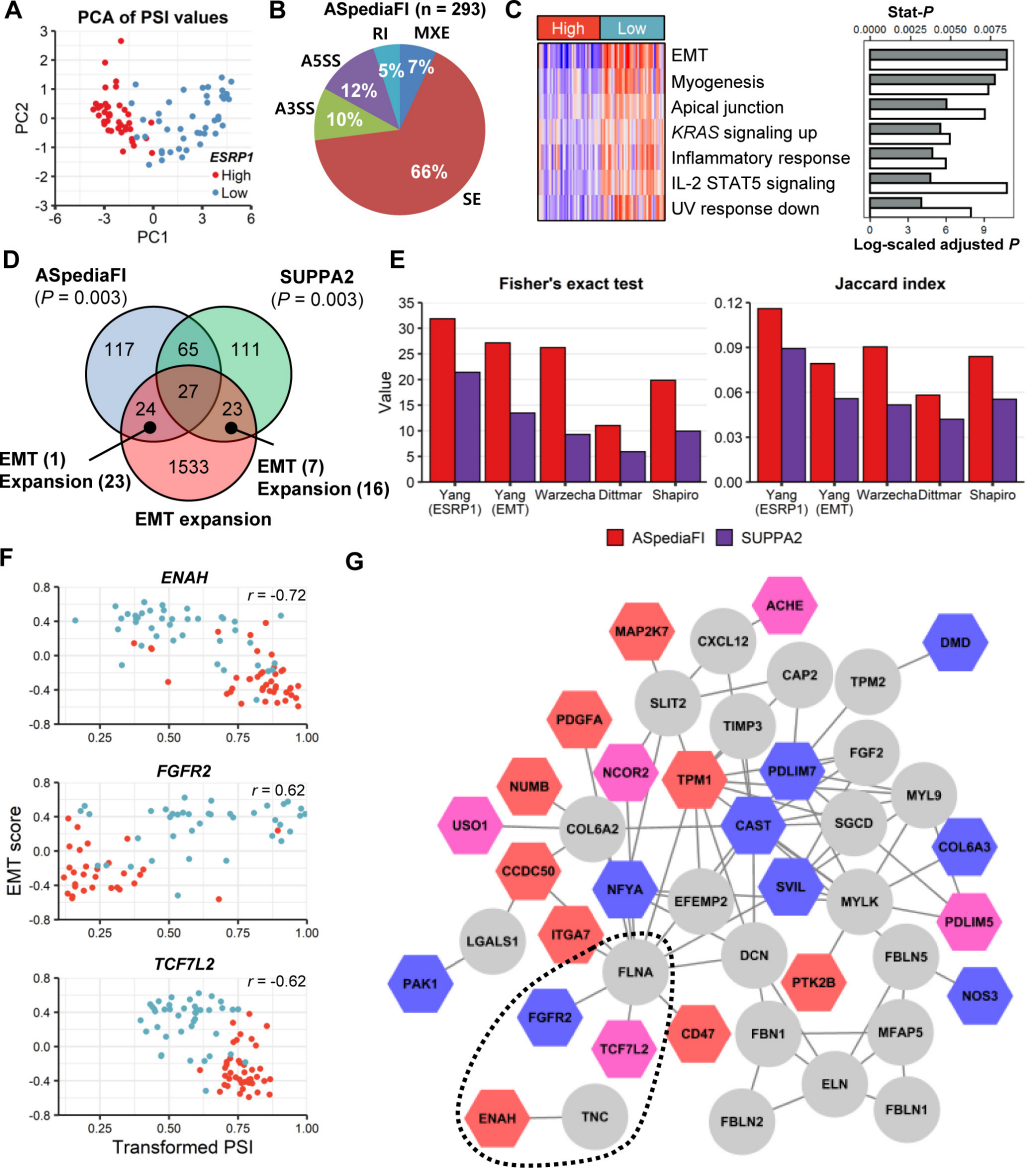
**Identification ofAS events associated with EMTpathway regulated by*ESRP1*** **A.** PCA scatter plot derived from PSI values of 293 DAS events. **B.** Percentage pie chart of five AS types. **C.** Pathway identification comparison between ASpediaFI and GSVA . Seven pathways in the heatmap row were chosen from the ASpediaFI pathway ranking, and columns were ordered by high and low groups. The heatmap demonstrates pathway-level GSVA scores estimated using gene expression profiles. The bar plot on the right presents both our stat-*P* values (gray) for pathway ranking and adjusted *P* values (−log_10_) of GSVA scores (white) comparing between *ESRP1*-high and *ESRP1*-low groups. **D.** Venn diagram of ASpediaFI, SUPPA2, and the EMT expansion gene set. *P* values for two AS sets denote enrichment with the EMT expansion gene set. **E.** Status bar plots to investigate AS event consistency identified by ASpediaFI and SUPPA2. Five EMT splicing gene signatures (Yang *ESRP1*[4], Yang EMT [4], Warzecha [57], Dittmar [6], and Shapiro [56]) were collected, and Fisher’s exact test *P* values (−log_10_) and Jaccard indices were calculated. **F.** Scatter plots between EMT pathway scores (y-axis) by GSVA and $\sqrt[{2}]{\mathrm{PSI}}$ (x-axis) for three AS events occurring in *ENAH*, *FGFR2*, and *TCG7L2*. Correlation coefficients (*r*) were added to each plot. Blue dots indicate *ESRP1*-low group and red dots indicate *ESRP1*-high group. **G.** A gene–AS interaction subnetwork identified by ASpediaFI. Circles denote gene nodes and hexagons denote AS event nodes. AS event nodes were filled in color by $\left|\Delta PSI\right|$ values. To extract smaller size EMT-relevant subnetwork for generating plot, we eliminated gene nodes belonging to the EMT expansion gene set with log_2_ FC < 2 and AS nodes with $\left|\Delta PSI\right|$ < 0.25. Multiple edges of one AS event node were trimmed except the one with the maximum score. The dotted ellipse indicates the interactions of three spliced genes in (F). EMT, epithelial–mesenchymal transition; FC, fold change.

The epithelial regulatory SF, *ESRP1*, is down-regulated during the EMT and plays a critical role in tumor progression [56], [69]. We analyzed the TCGA STAD dataset to examine *ESRP1*-related AS events and associated pathway regulation. The comparison analysis of the TCGA STAD level 3 RNA-seq dataset was restricted to SUPPA2 and our method. Samples were first classified into *ESRP1*-high (n = 41) and *ESRP1*-low (n = 42) groups based on *ESRP1* mRNA expression levels (RPKM). ASpediaFI identified seven pathways and 293 DAS events (Table S1) from a query gene set of 578 DEGs. The PSI profile of detected AS events illustrated the strong discriminatory power of our method (sensitivity, 100%; specificity, 69%; Figure 4A). The proportions of the five AS event types are presented in Figure 4B. SE (66%) was found to be three times as large as the sum of (22%) of A3SS and A5SS. Likewise, SUPPA2 detected 57% SE events, and the proportion was similar to our method. In pathway analysis, ASpediaFI ranked the EMT pathway on top and consequently identified EMT-associated pathways such as ‘myogenesis’ and ‘apical junction’ (Figure 4C, left). To further evaluate our pathway results, we compared our method with the gene expression-based GSEA method, GSVA (Figure 4C, right). The GSVA result also resembled our rankings except for two pathways, ‘IL2-STAT5 signaling’ and ‘UV response down’.

To investigate the association between AS events and EMT, we demonstrated enrichment with the EMT gene set as a gold standard gene set. The EMT pathway was chosen based on previous studies reporting that SF *ESRP1* serves as an upstream regulator of the EMT pathway [6], [56]. We compared the two results extracted from ASpediaFI and SUPPA2 with the EMT expansion gene set, including known and novel neighboring genes of the pathway (Figure 4D). The two results were equally enriched in the expansion gene set (Fisher’s exact test *P* < 0.003). Meanwhile, ASpediaFI (n = 23) exclusively identified more novel AS events than SUPPA2 (n = 16), as well as protein domain-associated events (ASpediaFI, 34.8% of 23 events; SUPPA2, 25% of 16; Table S4). We also compared two results with five AS sets for EMT studies or *ESRP1*-associated AS results derived from independent experiments and RT-PCR [4], [6], [56], [57]. The five sets were extracted from RNA-seq or SELEX-seq data for ESRP1-binding sites using *ESRP1* knockdown, siRNA, or EMT-inducing culture cells. Fisher’s test and Jaccard indices referring to five sets were notably better for the ASpediaFI DAS set than for SUPPA2 (Figure 4E).

In particular, ASpediaFI identified known EMT-associated AS events, *ENAH* SE, *FGFR2* MXE, and *TCF7L2* SE, which neither exist in the hallmark EMT pathway gene set nor were detected by SUPPA2. Three events were identified in all five EMT AS sets (Figure 4E). Additionally, PSI values of the three events were highly correlated (|*r*| = 0.62–0.72) with EMT pathway scores (Figure 4F). The three AS events belong to the EMT-associated subnetwork extracted by ASpediaFI (Figure 4G). Our network revealed the functional interactions of *TCF7L2* and *FGFR2* with the network hub *FLNA* [70]. The three AS events lead to changes in the protein domain involved in EMT [71], [72], [73]. *TCF7L2* SE is presented in the ‘N-terminal CTNNB1 binding’ domain, *FGFR2* MXE in the ‘Immunoglobulin I-set domain,’ and *ENAH* in the ‘EVH2 domain’ (Figure S5). *FGFR2* MXE generates two isoforms: *FGFR2*-IIIb, exclusive to epithelial cells, and *FGFR2*-IIIc, which causes a switch from the mesenchymal isoform and induces a change in ligand binding specificity, thereby regulating cell proliferation and differentiation (Figure S5) [72]. *TCF7L2* SE in the CTNNB1 binding domain affects the activity of Wnt/β-catenin target genes, and its deficiency was verified as the depletion of a proliferative cell compartment in the intestinal epithelium in mice [73]. Its switch-like exon usage is associated with invasive and mesenchymal-like breast tumors [71].

#### *Case study 3*: *AS events uncover neuronal development by RBFOX1 knockdown*

**Figure 5:**
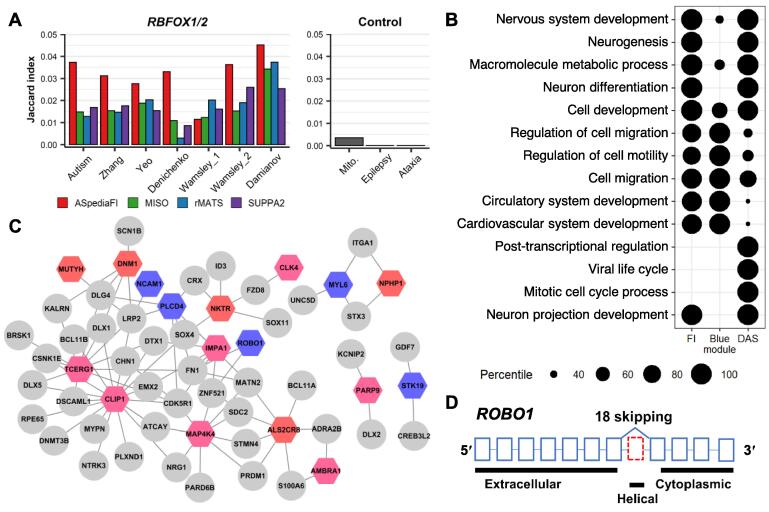
**Analysis of the *RBFOX1***-**knockdown RNA-seq dataset** **A.** Jaccard index bar plots to compare AS event consistency between four tools with previously identified *RBFOX1/2* induced splicing gene signatures [58], [59], [60], [61], [62], [63], [64]. Seven relevant *RBFOX1*/*2*-associated splicing gene sets were collected for benchmarking, and three sets for controls. **B.** Dot plot for percentile ranks of GO terms (row) from gene sets (column) by three different methods: our genes extracted by permutation *P* values (FI), neuronal development genes identified by WGCNA referring gene expression (Blue module), and DAS. The last two gene sets were derived from the previous study result [58]. **C.***RBFOX1*-associated subnetwork identified by ASpediaFI. To extract a smaller size subnetwork, we eliminated gene nodes belonging to neuron differentiation set with log_2_ FC < 0.25 and AS nodes with $\left|\Delta PSI\right|$ < 0.15. **D.** Exonic structure of exon 18 skipping (red) and protein domains of *ROBO1*. GO, Gene Ontology.

The spliced genes mediated by the RNA-binding protein RBFOX1 regulate neuronal development and are associated with developmental disorders such as autism [58], [74]. We analyzed the *RBFOX1*-knockdown RNA-seq dataset of primary human neural progenitor cells, including five *RBFOX1-*knockdown samples and five control samples. To be consistent with the previous study, we changed the reference pathway gene set to GO level 5 [58]. ASpediaFI identified 291 DAS events and nine pathways (Table S1) using a query gene set of 521 DEGs. For comparison with other methods, DAS events were aslo detected via MISO (n = 264), rMATS (n = 310), and SUPPA2 (n = 363). To verify the DAS results of the four methods, we computed the Jaccard index using seven relevant gene signatures (autism, n = 196 [58]; Zhang, n = 1103 [59]; Yeo, n = 1681 [60]; Denichenko, n = 63 [62]; Wamsley_1, n = 278 [63]; Wamsley_2, n = 580 [63]; and Damianov, n = 722 [64]) and three controls (Figure 5A). The seven relevant signatures were collected from previous splicing studies analyzing RNA-seq, CLIP-seq, or computational motif prediction to perform experiments for autism, human neuronal progenitors, or cortical interneuron cells [58], [59], [60], [62], [63], [64]. Controls were randomly selected from known gene sets: mitochondrial (n = 319), ataxia (n = 51), and epilepsy (n = 46). Our resultant AS sets exhibited higher similarity across the seven relevant sets than the control sets (Figure 5A).

Our pathway results identified neurogenesis, neuron differentiation, and nervous system developmental pathways (Table S1). To further evaluate the pathway detection performance, we compared our results ‘FI’ with two gene sets derived from a previous study [58], the ‘DAS’ set and co-expression subnetwork ‘Blue module’ gene set (Figure 5B). We chose the top five GO terms for each GSEA result from the three gene sets and computed their percentile ranks. GSEA using the ‘Blue module’ gene set failed to detect neuron differentiation and neurogenesis. The test results of the DAS set included discordant terms, such as post-transcriptional regulation and the viral life cycle. Unlike these two AS signature sets, the ASpediaFI result (FI) successfully identified the most relevant biological processes associated with neuron development in the top GO terms. This result illustrates the advantage of our integrative approach and the limitations of the previous approach (Blue module and DAS).

We identified an *RBFOX1*-associated module within a heterogeneous network (Figure 5C). The subnetwork included AS events of *ROBO1* and *CLIP1*, both of which had neural-regulated micro-exons (exons 3–27 nt) involved in an AS interaction network associated with an autism spectrum disorder in a previous study [74]. Among the AS events, three micro-exon events (*AP2M1*, *CLASP1*, and *ROBO1*) were identified as neural-regulated in a previous study. In particular, *ROBO1* exon 18 skipping induced helical domain exclusion and loss-of-function of ROBO1-SLIT2 signaling (Figure 5D). In our results, *ROBO1* exon exclusion was significant (permutation *P* value = 0.001, $\left|\Delta PSI\right|$ = −0.265; Table S1) and moderately correlated with the GSVA scores of the REACTOME ROBO receptor signaling pathway (*r* = −0.53).

## Discussion

The advent of next-generation sequencing led to the development of various novel methods for DAS analysis. Although DAS test methods have improved their accuracy, it is still challenging to interpret the biological relevance of resultant AS events. Here, we suggest an integrative method, ASpediaFI, to systematically identify AS events, co-expressed genes, and pathways together. ASpediaFI also provides functional interactions in the form of an interaction network. This enables users to understand co-expressed genes and global pathway activities regulated by splicing. Therefore, it helps to determine biologically relevant AS events.

To evaluate the performance of ASpediaFI, we analyzed a simulated dataset and compared it with three conventional analysis tools. We designed the sequencing depth conditions to cover read counts of 5 to 52 million and various sample sizes. In the simulation, a larger sample size and a higher sequencing depth dataset showed better performance for all the methods. When the conditions were unfavorable due to decreasing sequencing depth, ASpediaFI maintained better AUC values than the other tools (Figure 2B). However, ASpediaFI performance was reduced when working with a low sample size of less than 5. We could infer that the stability of ASpediaFI originates from our distinct approach for AS–gene correlation interaction network. Additionally, the correlation to establish a network restricts the minimum requirement of a sample size of over five for stable performance. It also showed an advantage in higher accuracy for lower sequencing depth samples. Our method is robust to various conditions of isoform usage (AS ratios). In short, our tool is executable for RNA-seq conditions with a minimum of 5 replicates per condition and a total read count of 5 million per sample.

Three case studies were performed to examine the performance of our tool and verify the biological relevance of the results. In case study 1, ASpediaFI enables the user to extract essential DAS results (n < 300), such as SUPPA2. However, MISO and rMATS dominantly detected SE type under the cutoff equal to SUPPA2, and fewer *de novo* event-comprised proportions (less than 20%). Therefore, it was unavoidable to select a more extensive DAS set (n > 2000) to include the five AS event types in the results. We showed that the two methods used to detect frequent SE types have the multiple-testing problem derived from tremendous test numbers, such as the DEG statistical test. As previously known, it is also accompanied by the burden of false-positive errors [16], [17]. Meanwhile, the other three methods perform independent test strategies for each AS type so that they cannot be pooled for all events. Hence, users should avoid equal *P* value cutoffs and consider separate DAS selection strategies for the characteristics of each method and each event type. Meanwhile, our results detected more RI-type events than the other three methods. Interestingly, 95% of RI in our results were exclusion events, and our AS type proportions were similar to the refined results of a previous analysis and TCGA studies, where A3SS and RI were detected as dominant event types [18], [19]. As previously known, Oxford Nanopore technology has demonstrated a great advantage of long-read sequencing (median read length of 712–948 bp) [68]. The dominance of our RI exclusion remarkably corresponded with that of Oxford Nanopore to be more precise in splicing analysis. In case study 2, AS event types identified from ASpediaFI exhibited similar proportions to SUPPA2 (Figure 4B). Here, we simplified the analysis for a two-condition human dataset, but our method can be extended to other complex conditions such as other organisms, time series, or multiple conditions.

ASpediaFI extracts pathways associated with AS regulation. Hence, we demonstrated the biological relevance of the resultant pathways and the AS event set. We compared our results with the conventionally used GSEA and GSVA methods, which refer to the gene expression profile. Case studies 1 and 2 presented a remarkable similarity between our study and the GSVA and previous studies [19], [72]. We also tested the enrichment of AS events with the HM pathway gene set (case 1) and EMT (case 2), considered as gold standard sets based on previous studies. Our method achieved superior performance compared to the other three DAS detection tools.

Additionally, we investigated the orthogonal evidence derived from RT-PCR. Among the identified results, splicing events of *SEP2*, *PFKM*, and *GCC2* were detected in all four methods and verified by RT-PCR [19]. The RI event in the *OGI* gene, an HM pathway neighbor, was detected only by ASpediaFI [75]. *PPOX*, an HM pathway member gene, was detected in all three methods except for SUPPA2. Interestingly, only ASpediaFI detected alpha-synuclein (SCNA), a member of the HM pathway. The splicing event of *SCNA* has been strictly evaluated in Parkinson’s disease, and oxidant-induced functional AS has been reported [76]. Our results showed higher reproducibility in the evaluation using AS events for case 2 than for SUPPA2 (Figure 4E). Moreover, *ENAH*, *FGFR2*, and *TCF7L2* (Figure 4F) identified in ASpediaFI are already evaluated in various studies as well as RT-PCR [71], [72], [73].

Considering both PSI and gene expression profiles, our approach offers a unique advantage in supporting integrative results. Instead of multiple tests for DAS, DEG, and GSEA, ASpediaFI systemically elucidates the interactions between AS and genes and delineates pathway regulation. In case 3, the previous *RBFOX1* study performed GSEA and network-based module identification for each DEG and DAS set [58]. This previous study employed a complex procedure from extracting large DAS sets (n = 996) to splitting AS sets into inclusion and exclusion groups. Moreover, their independent tests for extracting DEG and DAS could not elucidate the interaction between AS events or genes. In contrast, our method provides integrative results in a single analysis and detects neurodevelopmental pathways more precisely than previous results (Figure 4C).

Another novel characteristic of our method is to extract a small DAS set. In contrast, MISO and rMATS constitutively detect tremendous amounts of over a thousand AS events to extract biologically relevant events. Our method could extract fewer than 300 AS events to identify specific pathways in the three case studies. The minimal results provide the convenience of deciding subsequent evaluations such as wet-lab experiments. Meanwhile, our method requires various cutoffs and conditions to optimize the result. Here, we suggested 100–500 query gene set sizes based on three case studies. To test AS events of reliable read depth, the ‘low.expr’ option to mean minimum gene expression ensures enough read counts to detect precisely AS events. Actually, in case study 1, average median read counts of detected AS events were over 100. Furthermore, ASpediaFI supports numerous user-friendly features. Our method does not depend on any alignment tool and contains the minimum dependency of the input format. ASpediaFI refers to BAM files, gene models, gene interactions, or gene sets, which are widely used in gene expression analyses. This also supports a reasonable execution time. The time-consuming task of reading BAM files is eased with a multi-thread option, and the principal analysis step of DRaWR after preprocessing is executable with a computer (RAM 16 GB, CPU 3.40 GHz, and 5.23 min of execution time for the simulation dataset of sequencing depth 65× and sample size 20). Even though it was incomparable with ours because other methods could consume additional time for preprocessing steps, those methods took about 30 min (case 3) to 2 days (case 1) to analyze the same datasets.

The distinct characteristics of our method are limited. Additional reference requirements, such as interaction networks, could become a limitation. For instance, known gene interaction networks or pathway gene set information of new organisms are not available. In addition, our DAS results could be the indirect inference of DEG by the guilt-by-association because our tool extracts DAS similar to the DEG query. Thus, it could unexpectedly lead to an over-represented DAS effect. There remain several tasks to be implemented in future releases. Further studies include measuring the uncertainty of PSI values on low read count splicing regions, improving robustness to datasets of small sample size, and extending to cover *de novo* event types or novel experimental designs such as time-series must be performed.

## Conclusion

In this study, we developed a novel integrative approach, ASpediaFI, to extract AS–gene–pathway associations using a heterogeneous network. We verified the capability of our method to interpret the biological processes regulated by splicing. Based on simulation analyzed under various conditions, ASpediaFI showed superior performance to the other three DAS detection methods in terms of AUC metric. As shown in the three case studies, ASpediaFI successfully identified DAS events and biologically relevant pathways. We expect that ASpediaFI will be beneficial in providing insights into novel roles and global regulation of SFs and AS events.

## Code availability

ASpediaFI is supported as an open-source R package program. The tool, user manual, and case studies are publicly available at https://bioconductor.org/packages/ASpediaFI.

## CRediT author statement

**Kyubin Lee:** Formal analysis, Visualization, Software, Writing - original draft, Writing - review & editing. **Doyeong Yu:** Software, Formal analysis, Visualization, Writing - original draft. **Daejin Hyung:** Resources. **Soo Young Cho:** Supervision. **Charny Park:** Supervision, Conceptualization, Funding acquisition, Writing - original draft, Writing - review & editing. All authors have read and approved the final manuscript.

## Competing interests

The authors have declared no competing interests.

## Supplementary material

Supplementary Figure S1 **ROC curves comparing the performance of four DAS analysis tools using simulated datasets of varying sample sizes and****sequencing****depths** Benchmarking analyses were performed to measure the sample size effect (*n* = 20, 10, and 5 replicates per condition) and the sequencing depth effect (mean base coverage = 65×, 50×, 30×, and 15×). For each tool, the value of the AUC metric with its corresponding color is displayed in the legend (bottom right). The dashed line indicates a non-discriminative method that randomly guesses true DAS events, and the corresponding AUC value is 0.5.

Supplementary Figure S2 **Summary of AS type proportions identified by the four methods in Case study 1** Percentage pie charts of five AS types for (**A**) *SRSF2* and (**B**) *U2AF1* deficient MDS data are shown.

Supplementary Figure S3 **Precision and recall for HM and HM expansion gene set calculated from the three SFs deficient MDS dataset analysis using four DAS detection tools** The same resulting DAS genes from the comparison analysis (Figure 3D) were evaluated. For both the HM and HM expansion gene sets, ASpediaFI showed the highest precision value among the four tools at the cost of a relatively lower recall value. Each bar on the x-axis represents a DAS detection tool with its corresponding color at the legend (bottom).

Supplementary Figure S4 **Comparison of functional sequence features involved in DAS events identified by the four methods in Case study 1** Protein domains, nonsense mediated-decays, and isoform-specific protein–protein interactions features were retrieved from the ASpedia database for (**A**) *SRSF2* and (**B**) *U2AF1* deficient myelodysplastic syndrome datasets.

Supplementary Figure S5 **Exonic structures and protein domains of three EMT-associated AS events***ENAH* 11a SE induces the loss of partial ‘EVH2 domain,’ and FGFR2 MXE generates two isoforms on the ‘IgIII’ domain. *TCF7L2* 3a exon skipping on the ‘CTNNB1 binding domain’.

Supplementary Table S1 **ASpediaFI DAS analysis results for three case studies**

Supplementary Table S2 **Summary table of read counts generated in simulation analysis**

Supplementary Table S3 **ASpediaFI performance comparison according to correlation coefficient cutoffs in simulation analysis**

Supplementary Table S4 **Counts of AS events detected by ASpediaFI, MISO, rMATS, and SUPPA2 in Case study 1**

Supplementary Table S5 **ASpediaFI performance comparison under various query conditions for SF3B1 analysis in Case study 1**
