# Supplementary figures and images for: ASpediaFI: Functional Interaction Analysis of Alternative Splicing Events
Source: Genomics Proteomics Bioinformatics. 2022 Jan 25;20(3):466–82. doi: 10.1016/j.gpb.2021.10.004 (PMC9801047; doi:10.1016/j.gpb.2021.10.004)

**
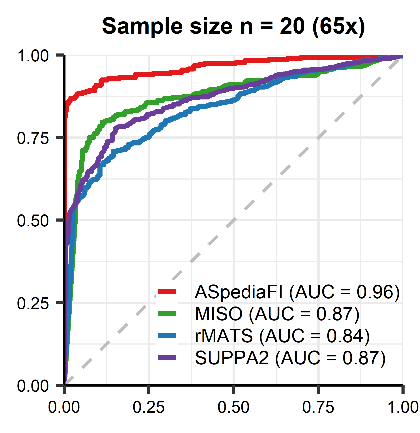

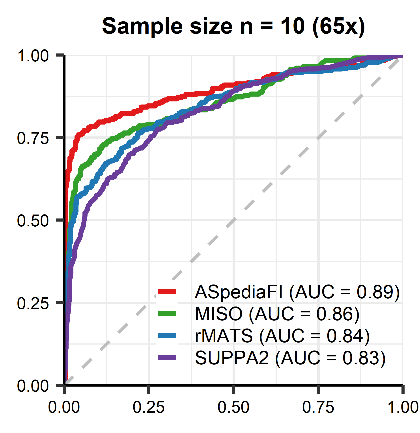

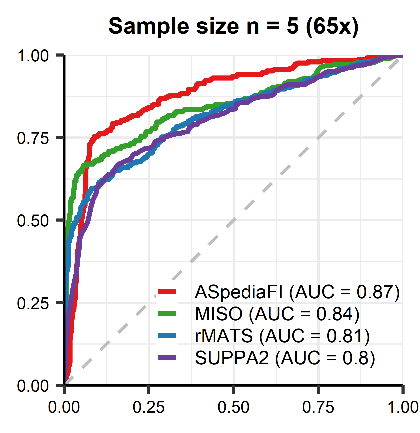
**

**
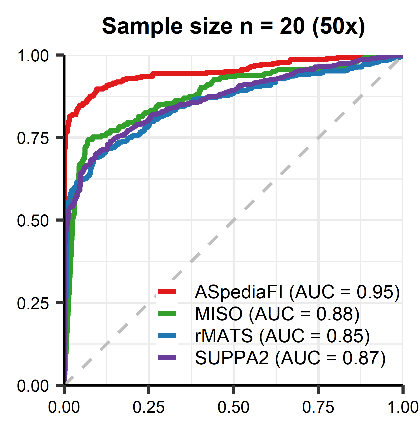

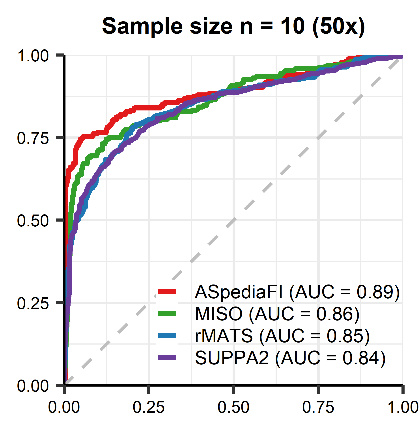

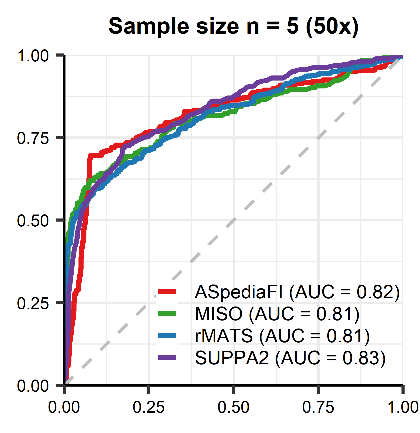
**

**
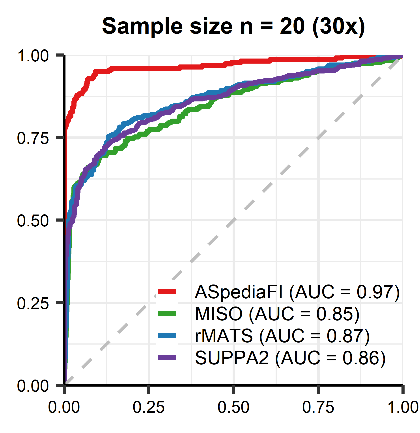

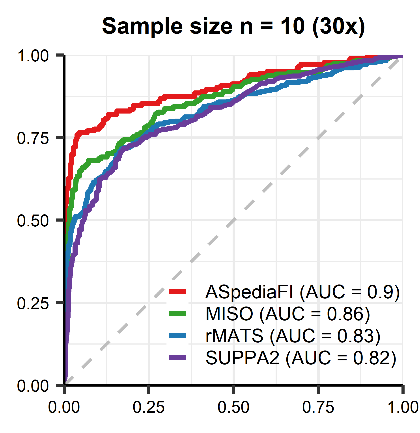

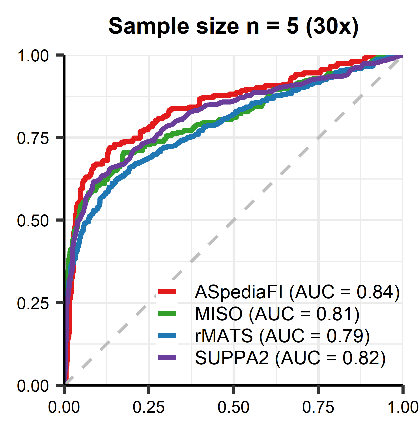
**

**
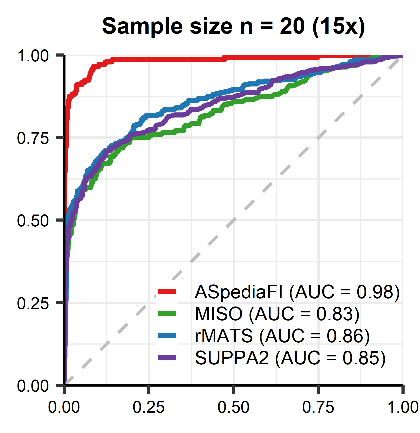

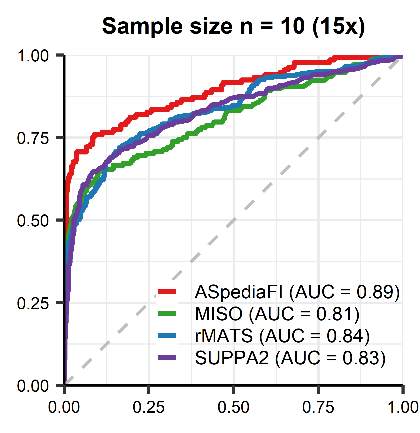

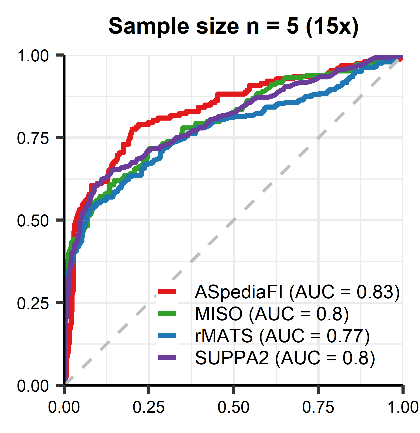
**

Supplement: Supplementary Figure S1 — ROC curves comparing the performance of four DAS analysis tools using simulated datasets of varying sample sizes andsequencingdepths Benchmarking analyses were performed to measure the sample size effect (n = 20, 10, and 5 replicates per condition) and the sequencing depth effect (mean base coverage = 65×, 50×, 30×, and 15×). For each tool, the value of the AUC metric with its corresponding color is displayed in the legend (bottom right). The dashed line indicates a non-discriminative method that randomly guesses true DAS events, and the corresponding AUC value is 0.5. [file mmc1.docx]

**
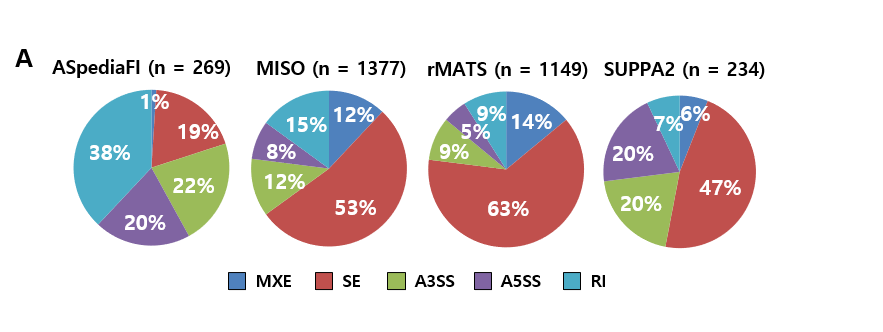

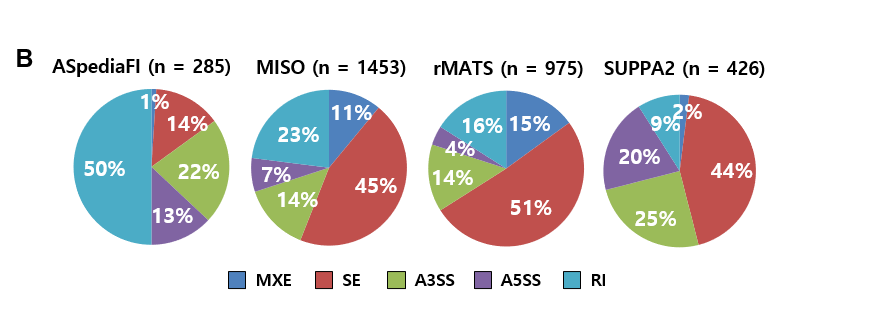
**

Supplement: Supplementary Figure S2 — Summary of AS type proportions identified by the four methods in Case study 1 Percentage pie charts of five AS types for (A) SRSF2 and (B) U2AF1 deficient MDS data are shown. [file mmc2.docx]

**
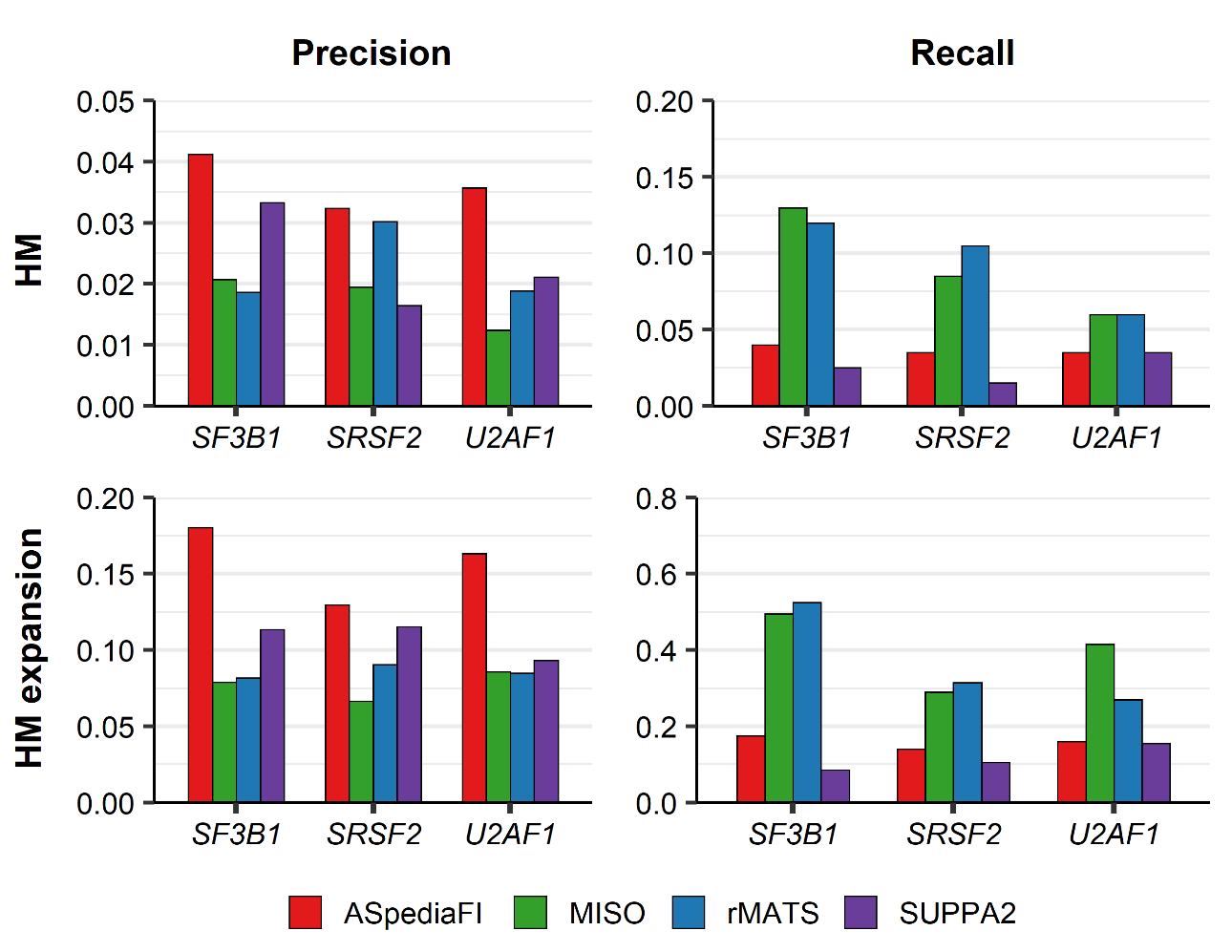
**

Supplement: Supplementary Figure S3 — Precision and recall for HM and HM expansion gene set calculated from the three SFs deficient MDS dataset analysis using four DAS detection tools The same resulting DAS genes from the comparison analysis (Figure 3D) were evaluated. For both the HM and HM expansion gene sets, ASpediaFI showed the highest precision value among the four tools at the cost of a relatively lower recall value. Each bar on the x-axis represents a DAS detection tool with its corresponding color at the legend (bottom). [file mmc3.docx]

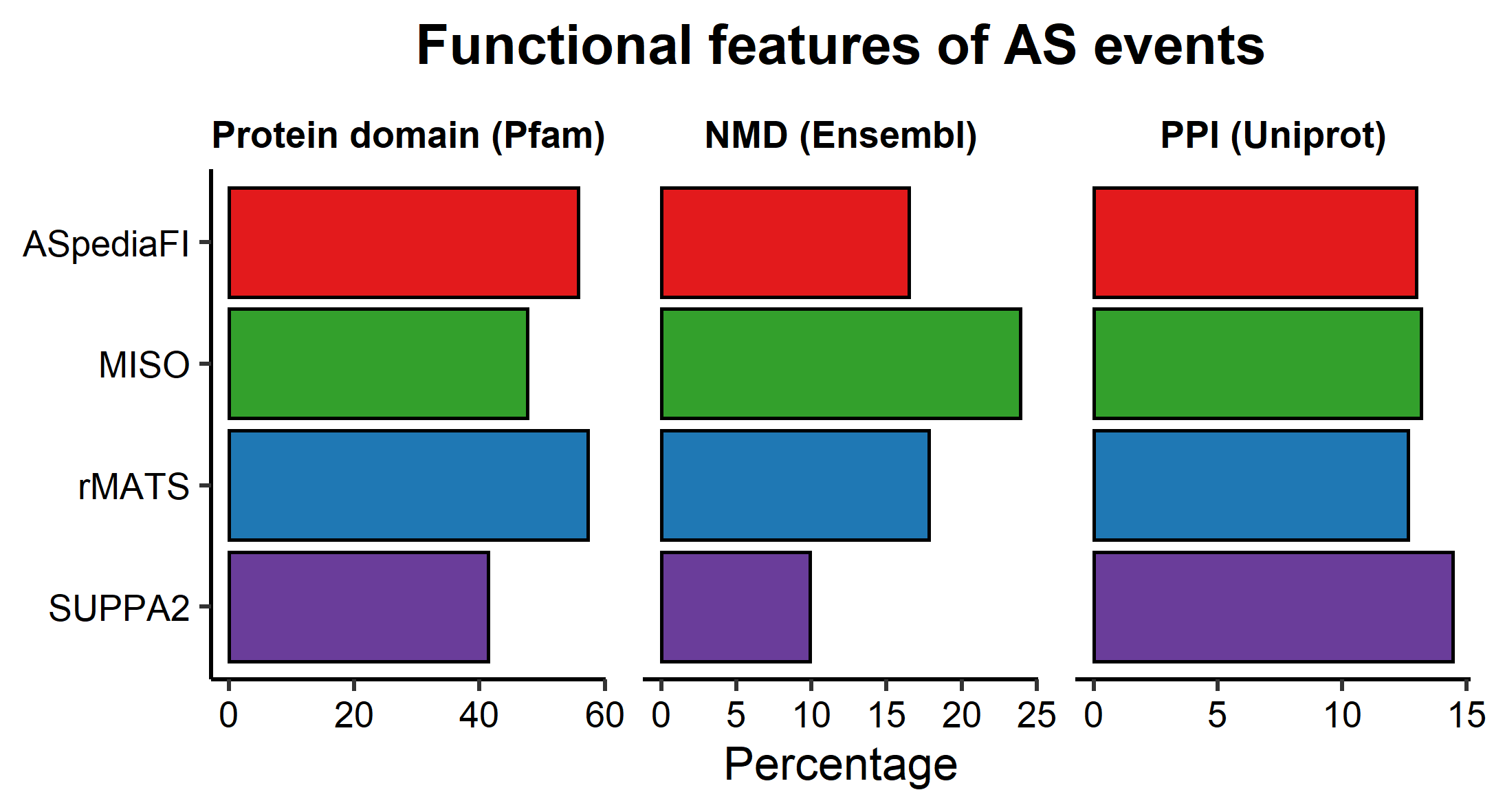


**B**


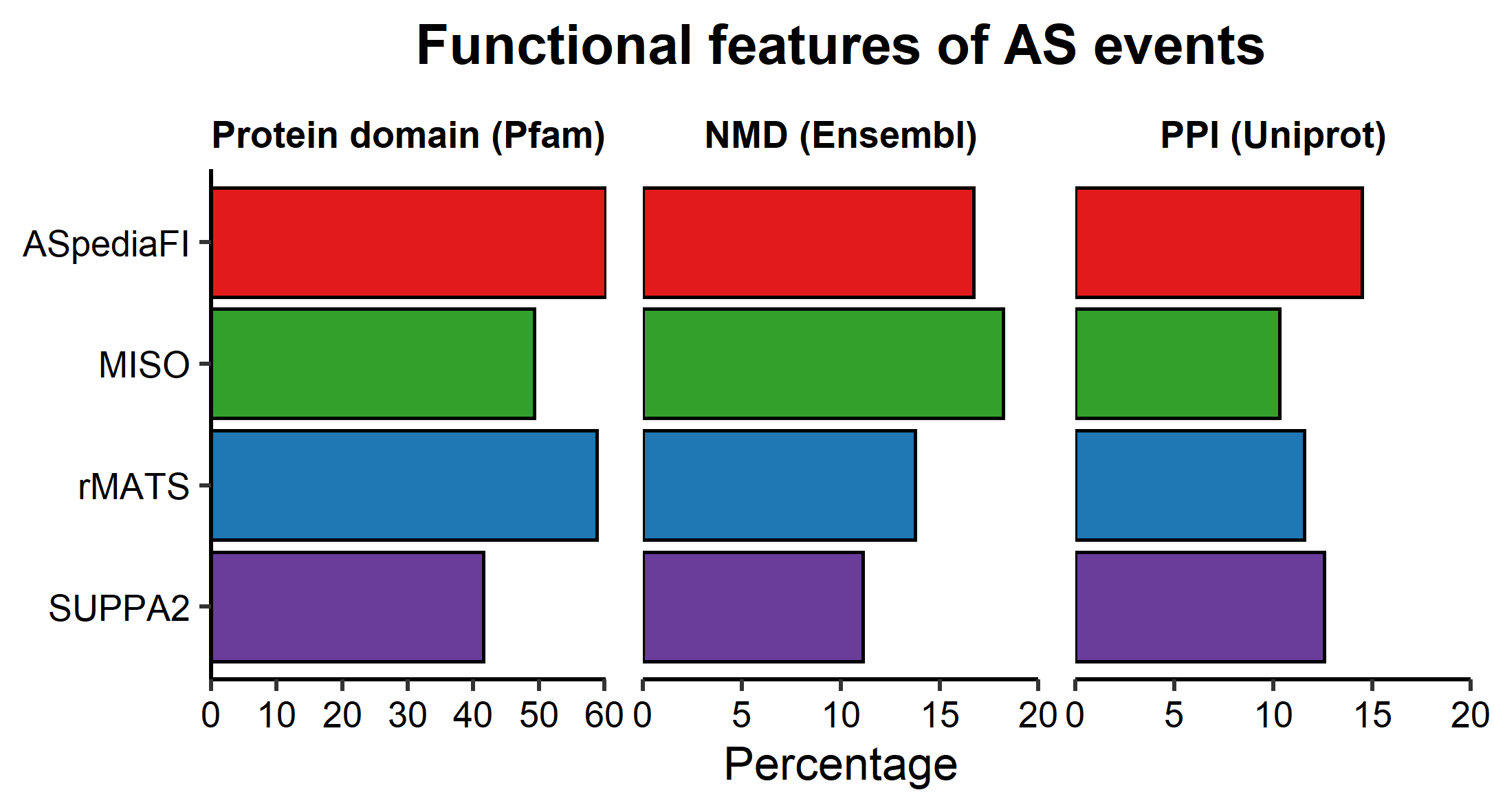


**A**

Supplement: Supplementary Figure S4 — Comparison of functional sequence features involved in DAS events identified by the four methods in Case study 1 Protein domains, nonsense mediated-decays, and isoform-specific protein–protein interactions features were retrieved from the ASpedia database for (A) SRSF2 and (B) U2AF1 deficient myelodysplastic syndrome datasets. [file mmc4.docx]
