# Supplementary material for: ASpediaFI: Functional Interaction Analysis of Alternative Splicing Events
Source: Genomics Proteomics Bioinformatics. 2022 Jan 25;20(3):466–82. doi: 10.1016/j.gpb.2021.10.004 (PMC9801047; doi:10.1016/j.gpb.2021.10.004)
Supplement: Supplementary Figure S5 — Exonic structures and protein domains of three EMT-associated AS eventsENAH 11a SE induces the loss of partial ‘EVH2 domain,’ and FGFR2 MXE generates two isoforms on the ‘IgIII’ domain. TCF7L2 3a exon skipping on the ‘CTNNB1 binding domain’. [file mmc5.docx]

**11a skipping**

**5’**

**3’**

**EVH1**

**LERER**

**EVH2**

**Pro-rich**

***ENAH***

**5’**

**3’**

***FGFR2***

**IIIb**

**IIIc**

**IgIII**

**TK**

**IgI**

**IgII**

**5’**

**3’**

***TCF7L2***

**CTNNB1 binding**

**3a skipping**

**HMG**
