# Supplementary material for: ASpediaFI: Functional Interaction Analysis of Alternative Splicing Events
Source: Genomics Proteomics Bioinformatics. 2022 Jan 25;20(3):466–82. doi: 10.1016/j.gpb.2021.10.004 (PMC9801047; doi:10.1016/j.gpb.2021.10.004)
Supplement: Supplementary Table S2 — Summary table of read counts generated in simulation analysis [file mmc7.docx]

**Table S2 Summary table of read counts generated in simulation analysis**

| Sample size | Depth 150× | Depth 65× | Depth 50× | Depth 30× | Depth 15× |
| --- | --- | --- | --- | --- | --- |
| 20 | 52,569,213 | 22,780,500 | 17,524,639 | 10,516,229 | 5,258,704 |
| 10 | 52,569,121 | 22,780,061 | 17,523,403 | 10,515,976 | 5,259,123 |
| 5 | 52,569,004 | 22,780,915 | 17,524,202 | 10,515,795 | 5,259,170 |

*Note*: The averages of the total read counts generated for each simulated RNA-Seq dataset are shown.
