# Supplementary material for: ASpediaFI: Functional Interaction Analysis of Alternative Splicing Events
Source: Genomics Proteomics Bioinformatics. 2022 Jan 25;20(3):466–82. doi: 10.1016/j.gpb.2021.10.004 (PMC9801047; doi:10.1016/j.gpb.2021.10.004)
Supplement: Supplementary Table S3 — ASpediaFI performance comparison according to correlation coefficient cutoffs in simulation analysis [file mmc8.docx]

**Table S3 ASpediaFI performance comparison according to correlation coefficient cutoffs in simulation analysis**

| **Depth** | **Sample Size** | ***r* > 0.4** | ***r* > 0.5** | ***r* > 0.6** |
| --- | --- | --- | --- | --- |
| **150×** | **20** | 0.94 | 0.96 | 0.97 |
|  | **10** | 0.91 | 0.90 | 0.88 |
|  | **5** | 0.83 | 0.82 | 0.83 |
| **65×** | **20** | 0.92 | 0.96 | 0.97 |
|  | **10** | 0.90 | 0.89 | 0.92 |
|  | **5** | 0.87 | 0.87 | 0.88 |

*Note*: AUC, area under the curve, values are summarized for each query condition.
