# Supplementary material for: ASpediaFI: Functional Interaction Analysis of Alternative Splicing Events
Source: Genomics Proteomics Bioinformatics. 2022 Jan 25;20(3):466–82. doi: 10.1016/j.gpb.2021.10.004 (PMC9801047; doi:10.1016/j.gpb.2021.10.004)
Supplement: Supplementary Table S4 — Counts of AS events detected by ASpediaFI, MISO, rMATS, and SUPPA2 in Case study 1 [file mmc9.docx]

| **Tool** | **AS event type** | ***SF3B1*** | ***SRSF2*** | ***U2AF1*** |
| --- | --- | --- | --- | --- |
| **ASpediaFI** | **A3SS*** | 66 (23.5%) | 60 (22.3%) | 64 (22.5%) |
|  | **A5SS*** | 28 (10.0%) | 53 (19.7%) | 37 (13.0%) |
|  | **SE*** | 33 (11.7%) | 50 (18.6%) | 40 (14.0%) |
|  | **RI*** | 151 (53.7%) | 102 (37.9%) | 142 (49.8%) |
|  | **MXE*** | 3 (1.1%) | 4 (1.5%) | 2 (0.7%) |
|  | **Total** | 281 | 269 | 285 |
| **MISO** | **A3SS** | 319 (15.1%) | 160 (11.6%) | 199 (13.7%) |
|  | **A5SS** | 159 (7.5%) | 111 (8.1%) | 96 (6.6%) |
|  | **SE** | 787 (37.1%) | 732 (53.2%) | 658 (45.3%) |
|  | **RI** | 656 (31.0%) | 203 (14.7%) | 340 (23.4%) |
|  | **MXE** | 198 (9.3%) | 171 (12.4%) | 160 (11.0%) |
|  | **Total** | 2,119 | 1,377 | 1,453 |
| **rMATS** | **A3SS** | 290 (12.1%) | 97 (8.4%) | 135 (13.8%) |
|  | **A5SS** | 141 (5.9%) | 59 (5.1%) | 38 (3.9%) |
|  | **SE** | 1009 (42.1%) | 729 (63.4%) | 497 (51.0%) |
|  | **RI** | 514 (21.5%) | 100 (8.7%) | 162 (16.6%) |
|  | **MXE** | 441 (18.4%) | 164 (14.3%) | 143 (14.7%) |
|  | **Total** | 2,395 | 1,149 | 975 |
| **SUPPA2** | **A3SS** | 76 (36.5%) | 48 (20.5%) | 105 (24.6%) |
|  | **A5SS** | 29 (13.9%) | 47 (20.1%) | 84 (19.7%) |
|  | **SE** | 66 (31.7%) | 110 (47.0%) | 190 (44.6%) |
|  | **RI** | 32 (15.4%) | 16 (6.8%) | 38 (8.9%) |
|  | **MXE** | 5 (2.4%) | 13 (5.6%) | 9 (2.1%) |
|  | **Total** | 208 | 234 | 426 |

**Table S4 Counts of AS events detected by ASpediaFI, MISO, rMATS, and SUPPA2 in Case study 1**

*Note*: The counts and proportions of the five AS, alternative splicing, types are summarized for the four tools and three SF, splicing factor, cases. * A5SS, alternative 5’ splice site; A3SS, alternative 3′ splice site, SE: skipping exon; MXE, mutually exclusive exons.
