# Supplementary material for: ASpediaFI: Functional Interaction Analysis of Alternative Splicing Events
Source: Genomics Proteomics Bioinformatics. 2022 Jan 25;20(3):466–82. doi: 10.1016/j.gpb.2021.10.004 (PMC9801047; doi:10.1016/j.gpb.2021.10.004)
Supplement: Supplementary Table S5 — ASpediaFI performance comparison under various query conditions for SF3B1 analysis in Case study 1 [file mmc10.docx]

| **Condition** | **Query size** | **Fisher’s *P*-value** | ***F_1_* score** |
| --- | --- | --- | --- |
| **DEG adj.*P*-value < 0.001 & logFC > 1** | 112 | 0.007 | 0.041 |
| **DEG adj.*P*-value < 0.001 & logFC > 0.6** | 498 | 0.072 | 0.030 |
| **DEG adj.*P*-value < 0.001 & logFC > 0.4** | 1111 | 0.174 | 0.025 |
| **Heme metabolism pathway** | 200 | 0.006 | 0.041 |
| **Random genes** | 100 | 0.587 | 0.015 |

**Table S5 ASpediaFI performance comparison under various query conditions for *SF3B1* analysis in Case study 1**

*Note*: Fishers’ exact test *P* values and *F_1_* scores to test the enrichment of heme metabolism pathway are summarized for each query set. The first DEG, differentially expressed gene, condition (adj. *P* value < 0.001 and log_2_ fold change > 1) was applied as the main query in case study 1.
